# Nanodiamond Regulated Electrolyte Enhances Thermal, Chemical and Structural Properties for Highly Reversible Zn Metal Anodes

**DOI:** 10.1002/advs.202516623

**Published:** 2026-01-12

**Authors:** Jiayan Zhu, Xuan Gao, Nan Gao, Shaoheng Cheng, Junsong Liu, Yuhang Dai, Zhengxiao Guo, Hongdong Li

**Affiliations:** ^1^ State Key Laboratory of High Pressure and Superhard Materials College of Physics Jilin University Changchun 130012 P. R. China; ^2^ Department of Chemistry The University of Hong Kong Pokfulam Road Hong Kong SAR 999077 P. R. China; ^3^ Thom Building Department of Engineering Science University of Oxford Oxford UK

**Keywords:** aqueous zinc‐ion battery, electrolyte engineering, heat generation, internal pressure, nanodiamond

## Abstract

Aqueous zinc‐ion batteries (AZIBs) suffer from inevitable internal resistance‐induced heat generation and competing hydrogen evolution, leading to high external cooling energy consumption and potential safety hazards. In this report, an electrolyte engineering strategy is proposed, involving nanodiamond (ND) additives to the commercial electrolyte. The ND particles assist in the reconstruction of the hydrogen bond network, reducing the desolvation energy of Zn^2+^, promoting the preferential deposition of (002) oriented Zn crystals, and effectively inhibiting water decomposition, Zn dendrite growth and heat‐induced side reactions. Importantly, compared to commercial counterparts, ND‐related electrolytes show relatively low impedance and high specific heat capacity (thermal conductivity), resulting in much reduced heat evolution and temperature rise. Such improvements are due to the key properties of nanodiamond, including its large specific surface area, abundant surface functional groups, and exceptional thermal conductivity. These collective enhancements not only minimize thermal and chemical side reactions but also reduce the internal pressure build‐up, as evidenced by only a 26% volume‐change in ND‐based pouch batteries, compared to a 76% rise with commercial electrolytes. Consequently, both coin cells and pouch batteries with the ND‐modified electrolyte exhibit much improved long‐term cyclability, specific capacity, rate capacity and coulombic efficiency, compared to those without NDs.

## Graphical abstract

Nanodiamond additives are dispersed in the aqueous electrolyte to organize water molecules, suppress gas evolution and metal corrosion, and guide zinc to deposit more uniformly. Together with enhanced thermal conductivity for fast heat removal, this strategy reduces temperature rise and degradation, enabling safer, more durable rechargeable zinc metal batteries in practical devices.

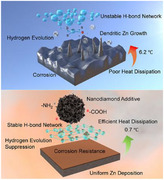

## 1 Introduction

Aqueous zinc‐ion batteries (AZIBs) show promising potential for energy storage due to their high safety, low cost, and environmental friendliness.^[^ ^1^ ^]^ Although the use of aqueous electrolytes reduces the risk of thermal expansion and explosion generally encountered in lithium‐ion batteries (LIBs) with organic solvents and alkali metals, commercial AZIBs still face the risk of thermal runaway, particularly at relatively large cell or pack sizes. As battery size increases, the surface‐to‐volume ratio decreases, which would reduce heat dissipation and increase the risk of localized overheating.^[^ ^2^ ^]^ Such a rise in temperature weakens the hydrogen bonds and accelerates water‐related side reactions (e.g., hydrogen evolution and corrosion), dendrite growth, and irreversible cathode dissolution.^[^ ^3^ ^]^ These effects compromise interfacial stability, increase internal resistance, and promote self‐discharge. In battery packs, the highly localized thermal instability can trigger cascading failure.^[^ ^4^ ^]^

The hydrogen evolution reaction (HER), in particular, leads to continuous generation and accumulation of hydrogen gas, which can destabilize the electrode–electrolyte interface, cause electrolyte leakage, or even result in cell rupture or explosion under confined conditions.^[^ ^5^, ^6^, ^7^ ^]^ Additionally, OH^−^ ions generated by HER react with Zn^2+^ to form insoluble by‐products such as Zn_4_SO_4_(OH)_6_·nH_2_O, further accelerating corrosion and degrading cycling performance.^[^ ^8^ ^]^ Extensive attempts of electrolyte and interface engineering have been carried out to regulate the solvation structure of Zn^2+^, to design functional interface coatings and capping agents,^[^ ^9^ ^]^ and to modify electrode structures and catalysts,^[^ ^10^, ^11^ ^]^ in order to inhibit side reactions. Recent additive‐focused studies have further improved interfacial stability and cycling in aqueous Zn systems.^[^ ^12^ ^]^ Progress has been achieved in promoting uniform zinc deposition, inhibiting dendrite growth, improving battery capacity and lifetime, and extending the operation window to low temperature/high temperature.^[^ ^13^, ^14^ ^]^ However, these traditional strategies usually overlook the heat evolution and local temperature/pressure rise during the cycling of AZIBs.

In principle, to reduce the temperature/pressure change in AZIBs, the strategies include lowering internal electric resistance, suppressing parasitic side reactions, and improving the thermal conductivity and specific heat capacity of the system.^[^ ^3^, ^4^ ^]^ Considering the practical demands of industrial applications, it is highly desirable to introduce modifiers or additives into the existing commercial components, without altering the production line, to improve the overall cost‐effectiveness of the battery. Among the fundamental components of AZIBs battery (anode, cathode, separator and electrolyte), electrolyte plays a crucial role in both transporting ions in battery and interacting with the electrodes and separator.^[^ ^15^ ^]^ Consistent with this view, recent work has shown that rational additive engineering can enhance ion transport and interfacial compatibility in AZIB electrolytes.^[^ ^16^ ^]^ Recent progress in aqueous Zn‐I systems: spanning I/Cl multi‐electron conversion, precipitated‐iodine cathodes, N‐rich MOF‐derived iodine hosts, Ni single‐atom electrocatalysis, self‐stratified biphasic electrolytes, and phase‐change thermal buffers—offers complementary routes to regulate halogen redox, stabilize interfaces, and manage heat, aligning with our goal of mitigating heat and pressure in practical AZIBs.^[^ ^17^, ^18^, ^19^, ^20^, ^21^, ^22^ ^]^ Moreover, it is the primary component and site for side reactions, determining battery performance.^[^ ^23^ ^]^ Hence, we focussed on electrolyte engineering with the aim to reduce heat generation and hydrogen evolution, thereby minimising internal temperature and/or pressure rise of AZIBs.

Regarding the selection of additives, it is accepted that the candidates must show chemical stability, compatibility with the electrolyte (separator and electrode), high thermal conductivity (heat capacity) and easy surface functionalization. Representative progress on additive screening and design for AZIB electrolytes has been reported, providing timely context for our additive choice.^[^ ^24^ ^]^ Among the various materials available, NDs stand out due to their unique properties such as super‐hardness, chemical inertness, functionalized surface carbon bonds (e.g., ─COO─ and ─NH), high specific surface area, and exceptional thermal conductivity,^[^ ^25^, ^26^ ^]^ which make NDs a highly promising additive in electrolytes. In Li^+^ and Na^+^ batteries, ND additive was used to enhance ionic transport, inhibit dendrite formation, increase battery specific capacity, and improve long‐term cyclability.^[^ ^27^, ^28^ ^]^ In recent years, ND has also been applied to AZIBs, mainly as a coating for Zn electrodes, which has improved the uniform deposition of Zn and inhibited Zn dendrite formation.^[^ ^29^, ^30^ ^]^ Recently, our group showed that an ND‐modified glass fiber separator improves the kinetics of Zn^2+^ transport.^[^ ^31^ ^]^ The additive of dimethyl sulfoxide (DMSO) and NDs to a zinc sulfate (2 M ZnSO_4_) electrolyte enhances the cycle life and coulombic efficiency of AZIBs.^[^ ^32^ ^]^ Nevertheless, the potential of functional materials (e.g., NDs) as electrolyte additives remains underexplored in advanced electrolyte engineering. Furthermore, critical challenges persist, including internal temperature regulation and pressure management in practical pouch cell configurations.

In this work, to suppress dendrite growth and side reactions while alleviating localized thermal accumulation, NDs are incorporated into a commercial aqueous electrolyte consisting of zinc sulfate (ZnSO_4_) and zinc trifluoromethanesulfonate (Zn(OTf)_2_) (abbreviated as ZS/ZF), resulting in a modified electrolyte designated as ND‐ZS/ZF. With relatively high specific heat capacity and thermal conductivity, the NDs are helpful for enhancing the thermal diffusion and suppressing the temperature rise. By addressing both electrochemical and thermal challenges, the ND‐ZS/ZF electrolyte significantly extends battery lifespan and performance. The ND‐ZS/ZF electrolyte provides an efficient solution that directly addresses the intertwined issues of interfacial instability, side reaction, by‐products and thermal effects in AZIBs, paving the way for more durable and efficient aqueous battery systems.

## 2 Results and Discussion

### 2.1 Physicochemical Properties of ND‐ZS/ZF Electrolyte

**Figure 1:**
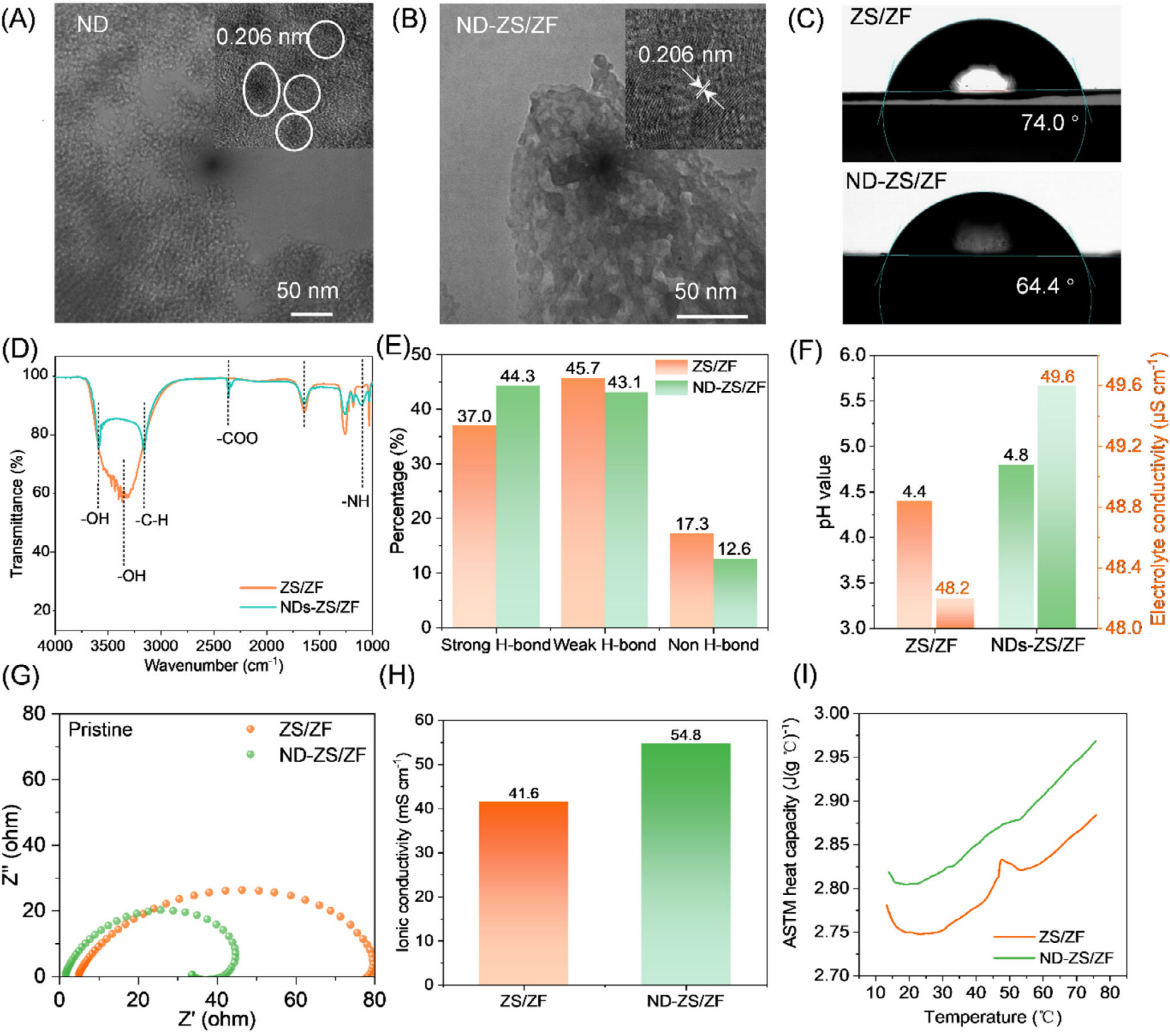
Physicochemical properties of the ND‐ZS/ZF electrolyte. A) TEM image of NDs. B) TEM image of NDs after immersion in ZS/ZF electrolyte. C) Contact angles on Zn foil. D) FTIR spectra of ZS/ZF and ND‐ZS/ZF electrolyte. E) Hydrogen‐bond distribution analysis. F) pH and ionic conductivity comparison. G) Nyquist plots of Zn||Zn cells. H) Ionic conductivity of different electrolytes of ZS/ZF and ND‐ZS/ZF. I) Specific heat capacity vs. temperature.

Detonation NDs (5–10 nm) were employed as an electrolyte additive in various concentrations (0–0.5 wt.%), with 0.1 wt.% yielding the optimal performance, and NDs dramatically reduce the Zn plating/stripping overpotential, indicating improved kinetic uniformity of Zn deposition (Figure S1, Supporting Information). High‐resolution transmission electron microscopy (HRTEM) confirmed stable diamond lattice fringes (0.206 nm, (111) plane) even after prolonged electrolyte immersion, indicating the structural robustness of the ND (**Figure** **1**A,B). This intrinsic stability is essential for their reliable use as thermally conductive fillers in extended battery operation. Contact angle measurements (Figure 1C) show improved wettability of ND‐ZS/ZF electrolyte on Zn foil (64.4°) compared to the unmodified ZS/ZF system (74.0°), suggesting enhanced electrolyte–electrode affinity.^[^ ^33^ ^]^

To elucidate the structural basis of such improvement, Fourier‐transform infrared (FTIR) spectroscopy was conducted (Figure 1D), revealing characteristic vibrational bands for ─NH, ─COO^−^, and ─C─H groups at 1200, 2400, and 3200 cm^−1^, respectively.^[^ ^31^ ^]^ Furthermore, the fitting curves (Figures 1E; S2, Supporting Information) reveal the chemical environments of water molecule O─H vibrations, including strong hydrogen bonds (3205 cm^−1^), weak hydrogen bonds (3410 cm^−1^), and nonhydrogen bonded species (3560 cm^−1^).^[^ ^34^, ^35^ ^]^ Notably, the proportion of strongly hydrogen‐bonded water increases significantly from 37.0% (ZS/ZF) to 44.3% (ND‐ZS/ZF), suggesting the ND surfaces—rich in hydroxyl and carbonyl groups—actively participate in and reorganize the electrolyte's hydrogen‐bond network, at least around the ND particles. This robust hydrogen‐bonding network, together with the presence of ─NH groups, reduces electrolyte–Zn interfacial tension,^[^ ^36^ ^]^ resulting in a lower contact angle and enhanced interfacial compatibility (Figure 1C). Notably, the abundant ─COO^−^ groups introduced by NDs also act as proton scavengers, leading to a moderate increase in electrolyte pH from 4.4 to 4.8 (Figure 1F). The decrease in free proton concentration weakens the competition between H^+^ and Zn^2+^ for electron transfer at the electrode, thereby improving Zn^2+^ transport and significantly enhancing the ionic conductivity of the electrolyte. Electrochemical impedance spectroscopy (EIS) measurements of Zn||Zn cells (Figure 1G) show a significant reduction in impedance for ND‐ZS/ZF electrolyte, with the ion conductivity of 54.8 mS cm^−1^ calculated by Equation S1 (Supporting Information) (Figure 1H), which is notably superior to other modified aqueous zinc electrolytes (Table S1, Supporting Information),^[^ ^37^, ^38^, ^39^, ^40^ ^]^ ensuring faster ion migration rates.

In addition to regulating the electrochemical environment, NDs significantly improve the thermal properties of the electrolyte. At room temperature (25 °C), the ND‐ZS/ZF electrolyte exhibits a specific heat capacity (*C_p_*) of 2.8116 J (g°C)^−1^, which is higher than that of the ZS/ZF electrolyte (2.7485 J (g°C)^−1^). According to the Equation S2 (Supporting Information), *C_p_* represents the heat absorbed per gram of electrolyte for a given temperature rise. The observed 2.3% increase in *C_p_* means the ND‐modified electrolyte can absorb more heat per unit mass, despite the small amount of ND added. This suggests that nanodiamonds enhance intermolecular interactions, such as hydrogen bonding, which require more energy to disrupt. Although the measured increase in thermal conductivity after ND addition appears modest (Figure 1I; Table S2, Supporting Information), rising from 0.5207 to 0.5252 W m^−1^ K^−1^ (0.9% increase), the enhancement in heat transfer is significant when considering the overall electrolyte performance. According to the rule of mixtures (Equation S3, Supporting Information), with an ND loading of only 0.1 wt.%, the predicted increase in thermal conductivity would be less than 1%. However, experimental results show that the thermal diffusivity of the ND‐ZS/ZF electrolyte increases from 0.1495 to 0.1682 mm^2^ s^−1^, corresponding to a 12.6% enhancement compared to the ZS/ZF electrolyte system. Such a boost is far beyond what a dilute additive would contribute by simple mixing, implying that the NDs improve heat transport not just by their own high thermal conductivity (diamond 5.2353 W m^−1^ K^−1^), but by altering the liquid structure. The net result is an electrolyte that can soak up and dissipate heat more efficiently, delaying the onset of temperature spikes during fast cycling.

### 2.2 Electrochemical Stability and Side Reaction Suppression

**Figure 2:**
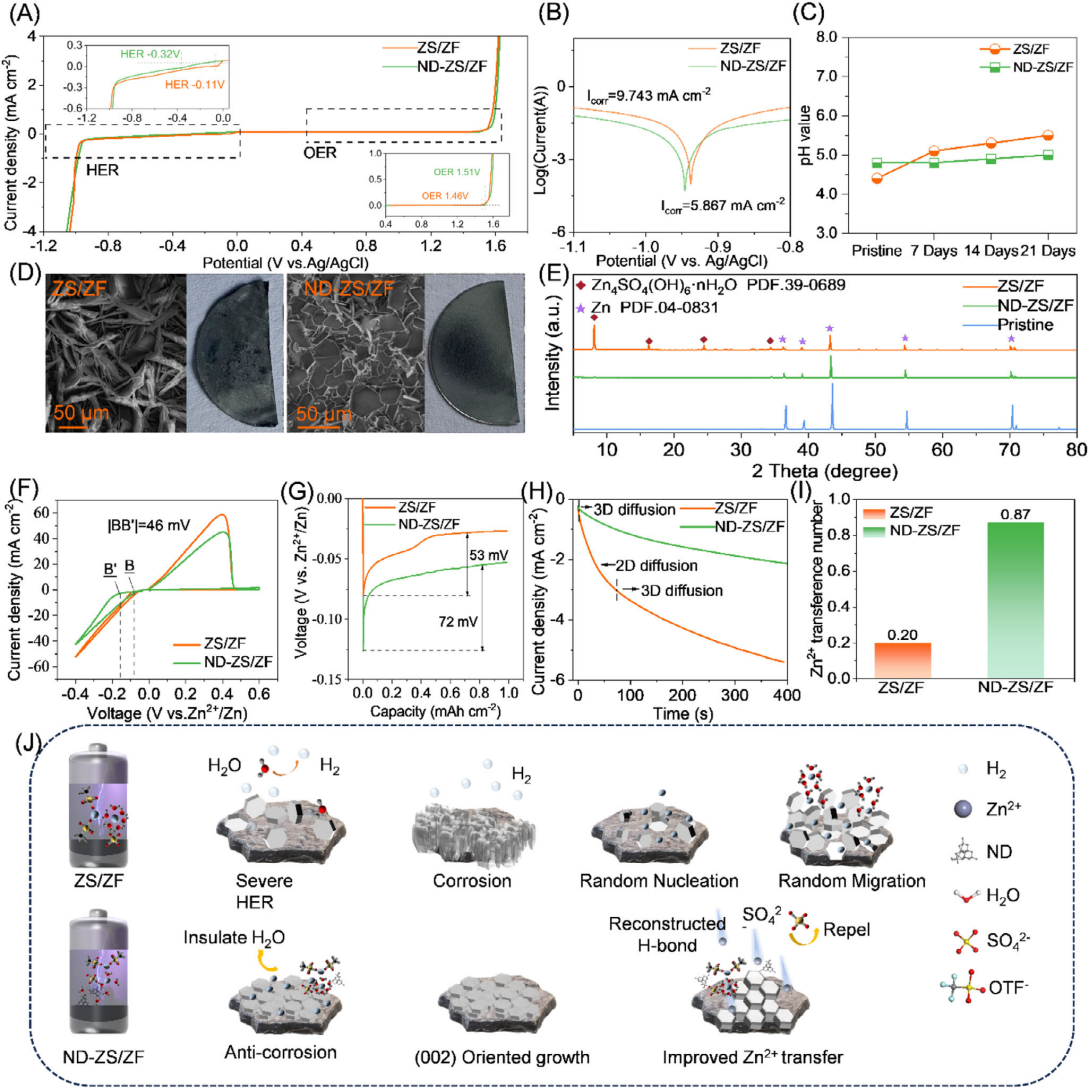
Electrochemical and morphological characterization of ZS/ZF and ND‐ZS/ZF electrolytes. A) ESW of different electrolytes. B) Tafel plots of the Zn anode in both ZS/ZF and ND‐ZS/ZF electrolytes. C) pH variation in ZS/ZF and ND‐ZS/ZF with Zn anodes immersed over different days. D) SEM images of Zn metal soaked in the ZS/ZF and ND‐ZS/ZF electrolytes. E) XRD pattern of Zn metal before and after immersion in both ZS/ZF and ND‐ZS/ZF for 21 days. F) Cyclic voltammograms of Zn||Cu cells. G) Galvanostatic charge–discharge profiles used to calculate the NOP for Zn plating/stripping in ZS/ZF and ND‐ZS/ZF electrolytes. H) CA test of Zn||Zn cells. I) Zinc ion transference number. J) Schematic illustrating the mechanism of ZS/ZF and ND‐ZS/ZF electrolytes.

The electrochemical stability window (ESW) test results (**Figure** **2**A) show that due to the highly correlated hydrogen bond network formed in ND‐ZS/ZF electrolyte, the HER of water molecules is significantly suppressed with decreasing HER potential from the conventional −0.11 to −0.32 V, while the oxygen evolution reaction (OER) potential is slightly elevated. As a result, the ESW width of ND‐ZS/ZF electrolyte reaches 1.83 V, which is not only larger than the width of ZS/ZF electrolyte in this work (1.57 V), but also far superior to the aqueous electrolytes reported in literature (Table S3, Supporting Information),^[^ ^40^, ^41^ ^]^ demonstrating excellent stability under both high and low potential conditions for ND‐ZS/ZF electrolyte. Tafel measurements (Figure 2B) show that the corrosion current density of the Zn anode in ND‐ZS/ZF electrolyte (5.867 mA cm^−2^) is ≈40% lower than in the ZS/ZF electrolyte (9.743 mA cm^−2^).Correspondingly, potential monitoring of Zn||Ti electrodes also reveals a substantial reduction in corrosion rate for ND‐ZS/ZF electrolyte (0.040 mg h^−1^), only 25% of that in ZS/ZF (0.156 mg h^−1^) (Figure S3 and Equation S4, Supporting Information). Although the electrolyte pH increases slightly from 4.4 to 4.8 (Figure 2C), the corresponding decrease in H⁺ concentration (from 3.98 × 10^−5^ to 1.58 × 10^−5^ mol L^−1^) alone is probably insufficient to account for such significant corrosion suppression.^[^ ^3^ ^]^ Further analysis by FTIR (Figure 1D) confirms abundant ‐COO^−^ and ‐NH groups on the ND surface, which can immobilize H^+^ ions via strong hydrogen bonding, substantially reducing their corrosive reactivity.

Long‐term immersion tests confirmed improved corrosion resistance in ND‐ZS/ZF electrolyte compared to ZS/ZF electrolyte. After 21 days, zinc foil immersed in ZS/ZF electrolyte formed significant Zn_4_SO_4_(OH)_6_·nH_2_O (ZHS) corrosion products due to H⁺‐induced reactions,^[^ ^42^ ^]^ increasing electrolyte pH (Figures 2C–E; S4, Supporting Information). Conversely, the ND‐ZS/ZF electrolyte maintained a smooth zinc surface, negligible by‐product formation, and stable pH levels, highlighting enhanced corrosion protection. With zinc foil immersed in ZS/ZF electrolyte after 21 days, the electrolyte resistance (R_e_) increased by a nearly 100‐fold from 5 to 461 Ω (Figure S5, Supporting Information), due to the continued corrosion of Zn anode, which results in consuming the electrolyte, forming porous ZHS by‐products, and hindering ion transport.^[^ ^43^ ^]^ Additionally, the low ionic conductivity of ZHS caused a significant increase in both the solid‐electrolyte interphase resistance (R_f_) and charge transfer resistance (R_ct_), ultimately resulting in a dramatic rise in interface impedance, thus shortening the cycle life of the zinc anode. Thanks to the anti‐corrosion properties of ND‐ZS/ZF electrolyte, the Zn||Zn symmetric cell achieved over 2500 h (104 days) of ultra‐stable cycle life in intermittent charge–discharge tests, far surpassing the 600 h (25 days) in the ZS/ZF electrolyte (Figure S6, Supporting Information).

Inhibiting zinc dendrite formation remains a critical challenge for aqueous zinc‐ion batteries, closely tied to zinc nucleation and growth characteristics. Cyclic voltammetry (CV) (Figure 2F) indicates that the nucleation overpotential (NOP) for zinc deposition in ND‐ZS/ZF is more negative than that in ZS/ZF, with the peak potential difference between the two electrolytes (|BB′|) reaching 46 mV. Correspondingly, galvanostatic charge–discharge profiles (Figure 2G and Equation S5, Supporting Information) nucleation overpotential (NOP) for ND‐ZS/ZF (53 mV) compared to ZS/ZF (72 mV), reflecting reduced interfacial energy barrier and enhanced plating/stripping reversibility.^[^ ^44^ ^]^ Chronoamperometry (CA) results (Figure 2H) further highlight the differences in zinc growth mechanisms: in the ZS/ZF electrolyte, rapid 2D diffusion dominates and persists for 70 s, resulting in uncontrolled lateral expansion of the deposition area and the formation of a porous zinc layer. In contrast, the ND‐ZS/ZF electrolyte quickly transitions to a steady‐state 3D diffusion regime, promoting uniform and compact zinc deposition. This suggests that uncontrolled growth of the porous zinc deposition layer leads to a rapid expansion of the specific surface area.^[^ ^45^ ^]^ In the ND‐ZS/ZF electrolyte, the 2D diffusion is suppressed and transfers to a constant 3D diffusion stage, and then a uniform zinc layer dominates the subsequent deposition. Optical and SEM imaging (Figures S7 and S8, Supporting Information) confirm that deposition capacities from 1 to 10 mAh cm^−2^ result in vertically grown, loose zinc clusters in the ZS/ZF electrolyte, but horizontally oriented, dense zinc layers (≈7.7 µm thickness) in the ND‐ZS/ZF electrolyte.

The ND‐ZS/ZF electrolyte featuring unique chemical coordination enables directed ion migration, and a significantly higher zinc ion transference number of 0.87 is realized, over four times that of the ZS/ZF electrolyte (0.20) (Figures 2I; S9 and Equation S6, Supporting Information). This enhancement is mainly attributed to the strong interaction between nanodiamonds and anions, which suppresses anion mobility and allows Zn^2+^ to dominate charge transport; further discussion on anion adsorption and solvation structure will be provided in the following section. The increased transference number results in the improvements of zinc ion conductivity and stability of zinc deposition, which can significantly enhance the safety and stability of the battery. Figure 2J provides a schematic illustration of the mechanisms underlying these improvements. The NDs in the ND‐ZS/ZF electrolyte facilitate the formation of a stable hydrogen bond network, which not only enhances the interaction between the electrolyte and the zinc anode but also reduces HER and corrosion. This restructuring also guides the uniform growth of zinc, improving ion transport and ensuring more stable cycling performance.

### 2.3 Mechanistic Insights Into the Enhanced Performance of ND‐ZS/ZF Electrolyte

**Figure 3:**
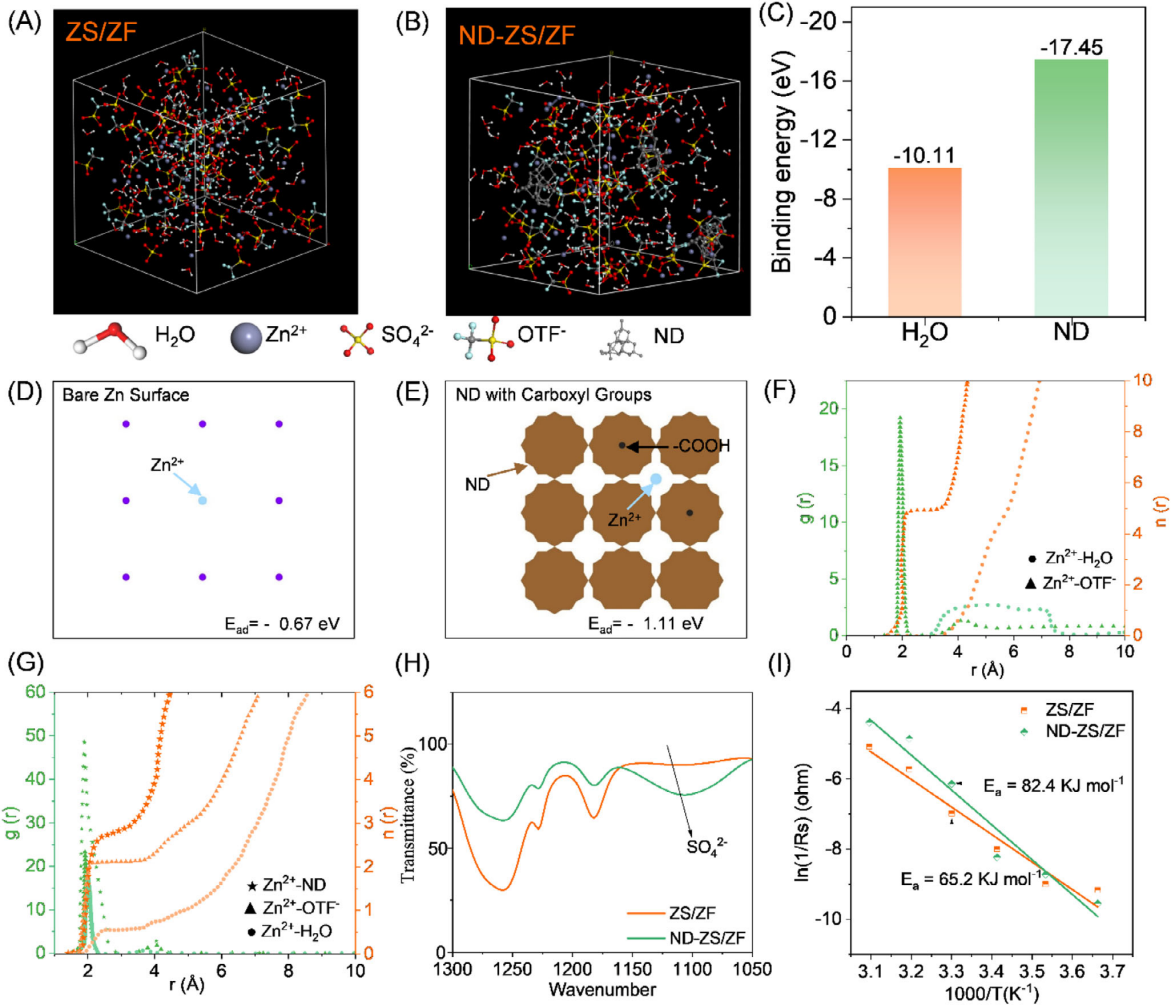
Molecular dynamics simulation and structural analysis of electrolytes. Solvation structure of Zn^2+^ in A) ZS/ZF and B) ND‐ZS/ZF electrolytes. C) Comparison of binding energy for Zn^2+^ in ZS/ZF and ND‐ZS/ZF electrolytes. Top‐views of simulation interaction and deposition of Zn^2+^ ions D) on bare Zn surface, E) on the Zn surface in the presence of ND‐modified electrolyte. RDF of Zn^2+^ in F) ZS/ZF and G) ND‐ZS/ZF electrolytes. H) FTIR spectra of ZS/ZF and ND‐ZS/ZF electrolytes. I) Thermal activation energy comparison for ZS/ZF and ND‐ZS/ZF electrolytes.

Molecular dynamics simulations of the solvation structure in both the ZS/ZF and ND‐ZS/ZF electrolytes, as shown in **Figure** **3**A,B, reveal key insights into the influence of NDs on the electrolyte properties. In the ZS/ZF electrolyte, Zn^2+^ ions predominantly form a relatively weak hydration shell with water molecules, which results in higher desolvation energy. This higher energy impedes efficient Zn^2+^ migration and contributes to uneven deposition during charge/discharge cycling. The ND‐ZS/ZF electrolyte benefits from the presence of NDs, which restructure the hydrogen‐bond network through interactions between the NDs' functional groups (such as ─COOH), water molecules and Zn^2+^ ions. This restructuring significantly lowers the desolvation energy, as evidenced by the binding energy (BE) of Zn^2+^ in the ND‐ZS/ZF electrolyte, calculated by Equation S7 (Supporting Information). The binding energy is 17.45 eV much lower than that of ZS/ZF electrolyte (−10.11 eV) (Figure 3C). The reduction in desolvation energy promotes a more stable solvation structure, facilitating efficient ion transport and ensuring uniform zinc deposition.^[^ ^46^, ^47^ ^]^

DFT calculations (Figure 3D,E) show that Zn^2+^ ions have a weaker adsorption energy on bare Zn (−0.67 eV) than on carboxyl‐functionalized nanodiamonds (−1.11 eV). A more negative adsorption energy means Zn^2+^ is more strongly attracted to the surface. Thus, with nanodiamonds present, Zn^2+^ ions are more likely to anchor onto many ND sites near the electrode, rather than clustering at a few spots on the bare Zn surface. This increases the density of nucleation sites, leading to a more uniform and finely distributed zinc deposition layer. Radial distribution function (RDF) analysis (Figure 3F,G) further supports this view, showing that Zn^2+^ ions in the ND‐ZS/ZF electrolyte exhibit sharper and more distinct peaks, indicating tighter coordination with water molecules. The influence of OTf^−^ anions is negligible due to the high dielectric constant of water.^[^ ^48^ ^]^ Overall, these results suggest that surface‐functionalized NDs can modulate Zn^2+^ interfacial behavior and contribute to improved deposition morphology.

Additionally, the diffusion coefficient (*D*) related to the particle radius and the viscosity based on the Stokes‐Einstein equation (Equation S8, Supporting Information) further corroborates the enhanced ion mobility and uniform deposition behavior in ND‐ZS/ZF electrolyte. This indicates a more ordered solvation structure presented in ND‐ZS/ZF electrolyte, leading to enhancing the ion mobility and deposition uniformity, which is crucial for improving the stability of the zinc anode during cycling. The strong interactions between NDs and water molecules significantly reduce the electrostatic interactions between water and Zn^2+^ ions, then the concentration of Zn^2^⁺ becomes high on the electrode surface, while the concentration of SO_4_^2−^ (Figure 3H) is simultaneously decreased.^[^ ^49^ ^]^ The desolvation energy (*E*_a_) was determined by fitting the impedance data as a function of temperatures (0–50 °C) according to Arrhenius Equation S9 (Supporting Information). As shown in Figures 3I and S10 (Supporting Information), the thermal activation energy (*E*_a_) for ND‐ZS/ZF electrolyte is 65.2 kJ mol^−1^, which is only 79% of the energy for ZS/ZF electrolyte (82.4 kJ mol^−1^). The reduction in activation energy suggests that the introducing NDs can efficiently reduce the energy barrier for ion transport and thermal‐induced side reactions.

### 2.4 Improved Stability and Thermal Properties of the Cells with ND‐ZS/ZF Electrolyte

**Figure 4:**
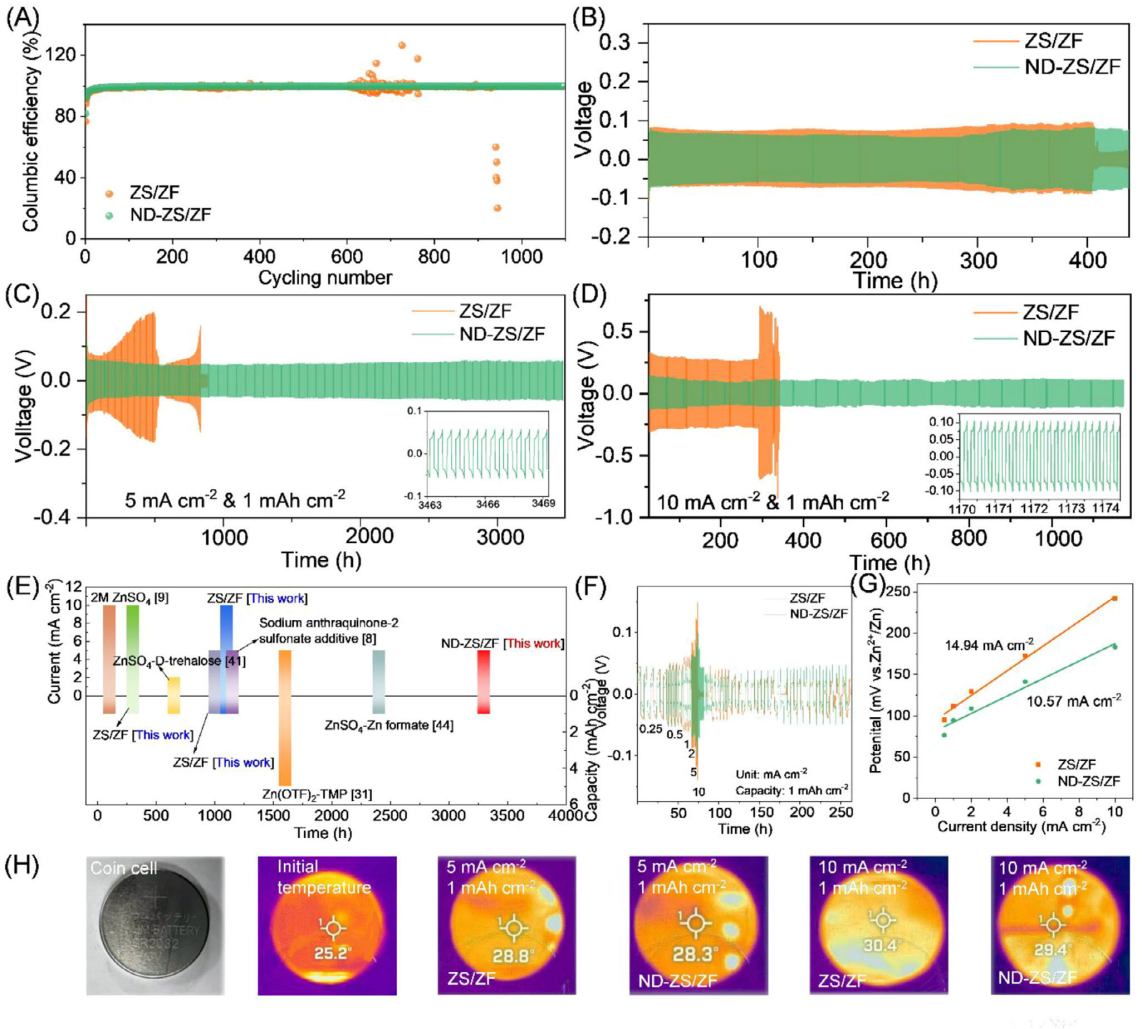
Electrochemical stability, thermal response, and dimensional evolution of coin cells with different electrolytes. A) Coulombic efficiency of Zn||Cu cells at 5 mA cm^−2^, 1 mAh cm^−2^. B) Zn||Zn battery at 80% depth of discharge. Long‐term cycling of Zn||Zn cells at C) 5 mA cm^−2^ and D) 10 mA cm^−2^. E) Comparison of corrosion current densities with previous reported electrolytes. F) Voltage polarization under varying current densities. G) Exchange current density. H) Surface temperature tested by infrared thermal imager after 100 cycles (bright spot due to camera lens reflection).

During charge–discharge cycles, AZIB temperature rise partly results from unavoidable intrinsic Ohmic heat. Excessive side reactions (e.g., zinc dendrite formation, electrolyte decomposition and zinc oxidation‐reduction) further elevate the electrolyte temperature, creating a self‐amplifying cycle of reactions and heat generation.^[^ ^50^ ^]^ Thus, minimizing heat and side reactions is crucial for AZIB safety and performance. The ND‐ZS/ZF electrolyte significantly enhances long‐term cycling stability, achieving a Coulombic efficiency of 99.82% after 1000 cycles (**Figures** **4**A; S11, Supporting Information). At high current density (6.5 mA cm^−2^) and 80% depth of discharge, the Zn||Zn symmetric cell with ND‐ZS/ZF electrolyte sustains stable cycling for over 400 h (Figure 4B), attributed to reduced polarization and suppressed side reactions under deep discharge conditions. As shown in Figure 4C, the Zn||Zn cell with ND‐ZS/ZF electrolyte achieves stable cycling for over 3300 h at 5 mA cm^−2^, whereas the conventional ZS/ZF electrolyte cell exhibits significant voltage fluctuations and fails after 1000 h. At an increased current density of 10 mA cm^−2^ (Figure 4D), the ND‐ZS/ZF electrolyte cell maintains stable voltage profiles for over 1100 h, compared to only 300 h with ZS/ZF electrolyte. Polarization analyses (Figure 4E) reveal significantly reduced voltage fluctuations and polarization in cells using ND‐ZS/ZF electrolyte across current densities from 0.25 to 10 mA cm^−2^, primarily due to suppressed Ohmic heating and surface side reactions by NDs. Furthermore, the exchange current density for ND‐ZS/ZF electrolyte, estimated at 48.59 mA cm^−2^ (Figure 4F,G and Equation S10, Supporting Information), confirms improved Zn deposition kinetics.

In Figure 4H, coin cell temperature tests (Figure S12, Supporting Information) reveal notable heat accumulation at high current densities. After 100 cycles at full charge, the ZS/ZF electrolyte cell temperature rises from 25.2 to 30.4 °C, whereas the ND‐ZS/ZF electrolyte limits the increase to 29.4 °C, highlighting improved thermal regulation due to the nanodiamond additive. Although elevated temperatures enhance zinc‐ion diffusion (Stokes–Einstein equation, Equation S8, Supporting Information), excessive ion aggregation at high current density exacerbates uneven deposition and heat generation.^[^ ^51^ ^]^ Pressure analysis (Figure S13, Supporting Information) shows pronounced swelling of ZS/ZF cells from 3.10 to 3.16 mm (5 mA cm^−2^) and 3.22 mm (10 mA cm^−2^), representing expansions of 12.06% and 24.12%, respectively, driven by hydrogen gas accumulation and internal pressure buildup. In contrast, ND‐ZS/ZF cells exhibit minor expansions to 3.12 and 3.13 mm, corresponding to only 4.02% and 6.03% increases, demonstrating suppressed gas evolution. Postcycling morphology confirms this advantage: ZS/ZF electrolyte results in a porous zinc deposition (20 µm thick) predominantly oriented along the (101) plane (Figure S14, Supporting Information), prone to short‐circuit risks. Conversely, the ND‐ZS/ZF electrolyte forms a dense, uniform zinc layer (8 µm thick) with preferred (002) orientation (Figure S15, Supporting Information), significantly improving mechanical stability and interfacial robustness.

### 2.5 Thermal‑Stable Zn||VO_2_ Coin Cells with ND‑ZS/ZF Electrolyte

Cycling stability and thermal management capability are the two key challenges in practical applications for AZIBs. Compared with the in‐depth studies related to cycling stability, the discussions on heat‐induced degradation and side reactions are often overlooked. In the conventional electrolytes, side reactions/by‐products such as HER, dendrite growth, and corrosion on Zn anode, generally lead to rapid capacity fading and excessive heat accumulation, which results in severely limiting practical applications.^[^ ^15^ ^]^ NDs with unique surface characteristics offer abundant active sites that can adsorb zinc ions and stabilize their deposition pathways, and effectively reduce the localized hotspots on electrode surface.

**Figure 5:**
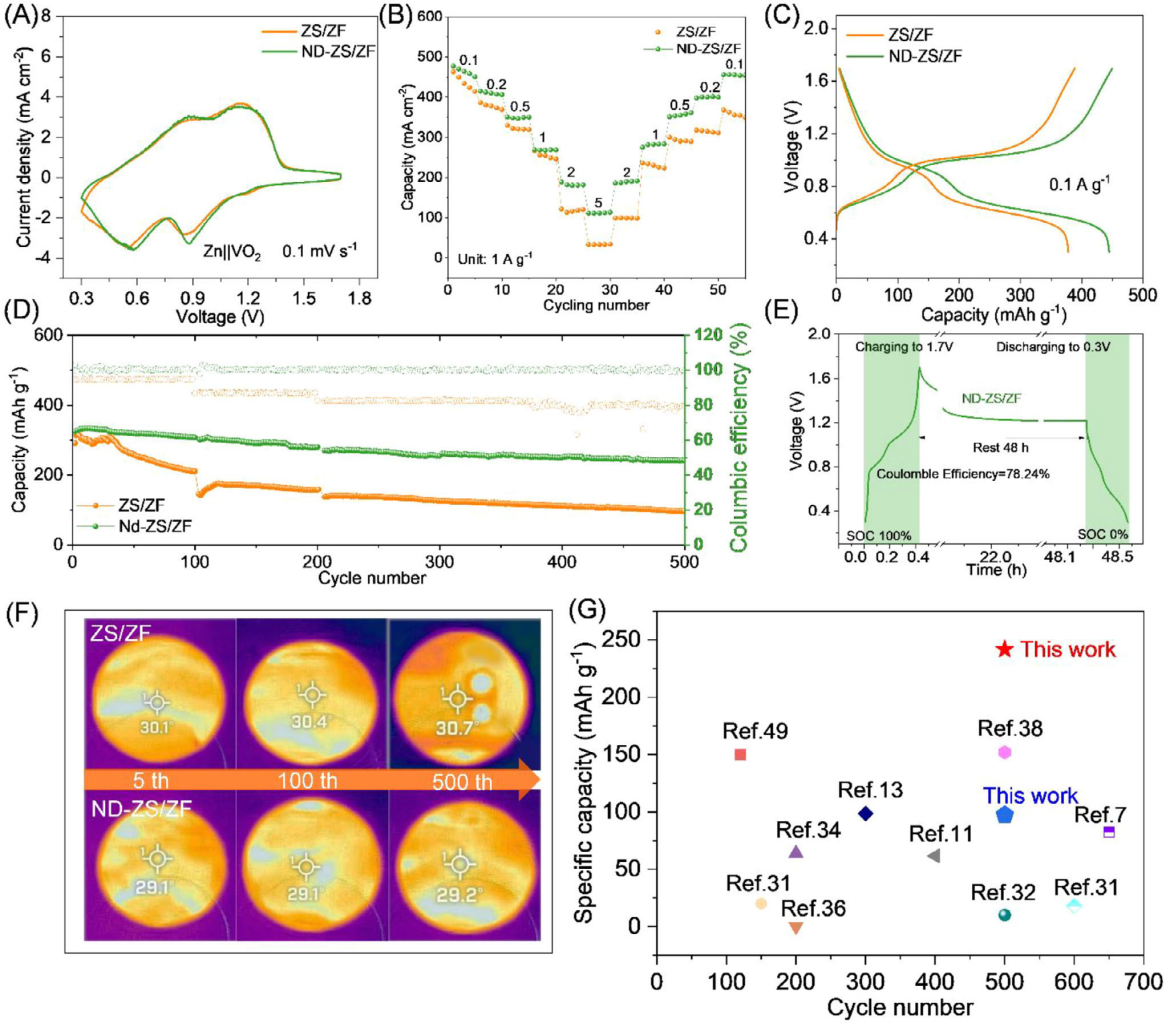
Electrochemical performance, stability, and thermal behavior of Zn||VO_2_ coin cells with ZS/ZF and ND‐ZS/ZF electrolytes. A) CV curves at 0.1 mV s^−1^. B) Rate capability of Zn||VO_2_ cells with ZS/ZF and ND‐ZS/ZF electrolytes. C) Galvanostatic charge–discharge curves at 0.1 A g^−1^. D) Long‐term cycling performance and corresponding coulombic efficiencies obtained at the current of 1 A g^−1^. E) Self‐discharge performance. F) Thermal images after charging for varying cycling processes. G) Comparison of cycling stability and specific capacity. For clarity, the rate‐capability values in panel (B) are taken from early cycles after stabilization at each rate, whereas the cycling data at the same nominal current density (e.g., 1 A g^−1^ in panel (D)) represent aged states during prolonged continuous operation.

For the Zn||VO_2_ coin cell applied with ND‐ZS/ZF electrolyte and ≈6.5 mg cm^−2^ active material loading VO_2_ cathode, as observed in CV curves (**Figure** **5**A), the electrode polarization is slightly increased, and the electrochemical behaviour shows stable Zn^2+^ insertion and extraction. In Figure S16 (Supporting Information), the CV tests at varying scan rates of 0.1–0.5 mV s^−1^ demonstrate the factor that the electrochemical reaction in Zn||VO_2_ coin cells is governed by both ion diffusion and pseudocapacitance.^[^ ^52^ ^]^ The ND‐ZS/ZF electrolyte shows a higher pseudocapacitance contribution (Equation S11, Supporting Information), indicating that the electrode materials achieve more efficient charge and discharge cycles in a shorter time attributed to the assistance of NDs, thus enhancing the battery's energy and power densities (Figure 5B). Long‐term cycling tests reveal that at a current density of 1 A g^−1^ (Figure 5C), the coin cell with ND‐ZS/ZF electrolyte maintains 74.32% capacity retention after 500 cycles, whereas the ZS/ZF electrolyte cell only retains 33.41% (Figure 5D). Consequently, the ND–ZS/ZF electrolyte yields a higher discharge plateau and a narrower charge–discharge voltage gap in the Zn||VO_2_ cell, indicating significantly reduced polarization due to improved Zn^2+^ transport and faster interface kinetics.

Self‐discharge tests emphasize the effectiveness of NDs in mitigating parasitic reactions. After 48 h, the ND‐ZS/ZF cell maintains a significantly higher coulombic efficiency (78.24%) compared to the ZS/ZF cell (59.49%) (Figures 5E; S17, Supporting Information), underscoring the role of NDs in preserving active materials by suppressing gas evolution, dendrites, and oxidative decomposition. Thermal imaging results (Figure 5F) reveal that after 500 cycles and upon reaching the fully charged state, the ZS/ZF‐based cell reaches a surface temperature of 30.7 °C, representing a 5.5 °C increase from its initial state (25.2 °C). The ND‐ZS/ZF‐based cell shows a milder increase to 29.2 °C (risen by 4 °C), indicating a relatively better thermal regulation. Correspondingly, internal pressure measurements (Figure S18, Supporting Information) indicate minimal swelling (1.2%) for the ND‐ZS/ZF cell, vs significant expansion (4.6%) for the ZS/ZF cell due to parasitic gas formation. These thermal and pressure differences correlate with polarization‐induced Joule heating and elevated interfacial impedance, further evidenced by pronounced voltage hysteresis (Figure S19, Supporting Information) and higher resistances (R_b_, R_sei_, R_ct_, Warburg impedance (W)) revealed by EIS analyses (Figure S20; Table S4, Supporting Information). In particular, the R_b_ value of the ND‐ZS/ZF cell is as low as 2 Ω, only 16.7% of that of the ZS/ZF cell (12 Ω), demonstrating its remarkable capability in suppressing internal resistance and Joule heat generation. The specific capacity versus cycle number for various aqueous Zn‐ion battery systems reported in literature is summarized in Figure 5G indicating that the system developed in this work (green pentagram) achieves a superhigh specific capacity (241.7 mAh g^−1^) after long‐term cycles, which highlights the superior long‐term cycling performance and capacity retention enabled by the proposed electrolyte engineering. The above findings mean that the coupling and feedback among many factors and processes (such as parasitic side reactions, thermal effect, internal impedance/pressure, etc) can be successfully addressed by simply introducing NDs into electrolyte system.

### 2.6 Practical Demonstration in Pouch Cells

**Figure 6:**
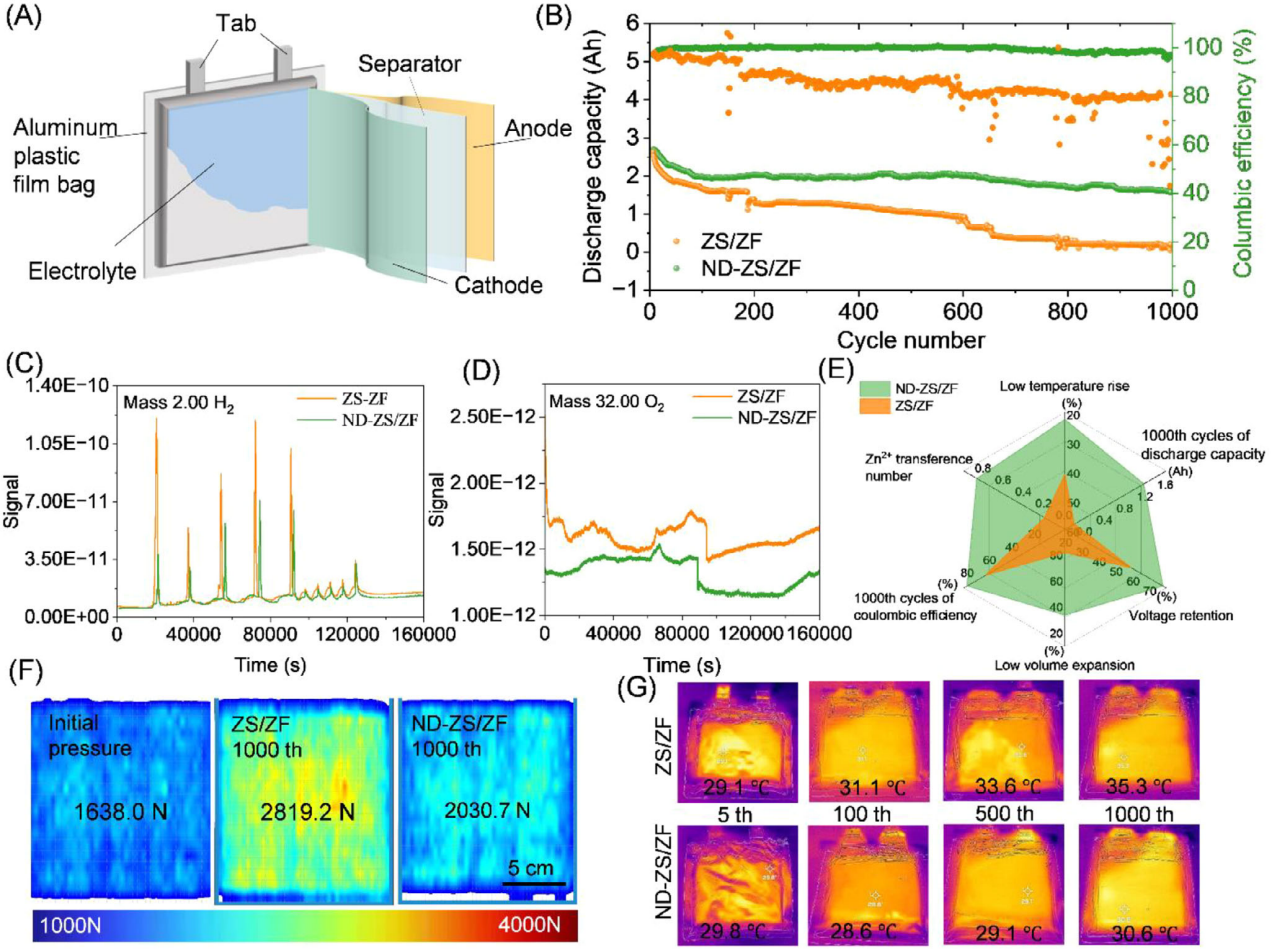
Comprehensive evaluation of the cycling performance, gas evolution, internal pressure, and thermal behavior of Zn||VO_2_ pouch cells using ZS/ZF and ND‐ZS/ZF electrolytes. A) Schematic illustration of the soft‐pack battery configuration. B) Long‐term cycling performance and coulombic efficiency after 1000 cycles. C,D) DEMS profiles of hydrogen and oxygen evolution. E) Radar chart comparing key performance indicators after 1000 cycles. F) Internal pressure distribution comparison before and after cycling. G) Infrared thermal images showing the elevated temperatures of pouch cells.

The practical potential of ND‐ZS/ZF electrolyte was validated using 2.6 Ah pouch cells with four‐anode and three‐cathode layer configurations (**Figures** **6**A; S21, Supporting Information). Long‐term cycling tests (Figure 6B) revealed that ND‐ZS/ZF pouch cells retained over 60% capacity and a stable ≈80% Coulombic efficiency after 1000 cycles, significantly outperforming control cells using conventional ZS/ZF electrolyte, which retained less than 5% capacity despite similar Coulombic efficiency. The ZS/ZF control undergoes functional failure at 197 cycles, evidenced by a sharp capacity collapse and erratic Coulombic efficiency thereafter. This instability arises from dendrite‐induced soft shorts and parasitic HER/OER, as supported by the stronger H_2_/O_2_ signals (Figure 6C,D), higher internal pressure (Figure 6F), and larger temperature rise (Figure 6G). Data beyond this point are not considered reversible capacity. Differential electrochemical mass spectrometry (DEMS) analysis (Figure 6C) showed substantially lower hydrogen evolution in ND‐ZS/ZF electrolyte, particularly during initial cycles, attributed to stable ND‐enhanced hydrogen‐bond networks promoting uniform zinc deposition and suppressing dendrite growth. Conversely, the ZS/ZF system displayed pronounced hydrogen evolution due to unstable electrolyte‐anode interfaces causing uneven zinc deposition and enhanced water decomposition. Additionally, ND‐ZS/ZF electrolyte effectively reduced cathode‐side oxygen evolution (Figure 6D), confirming comprehensive bidirectional interface stabilization. DEMS signals for CO and CO_2_ (Figure S22, Supporting Information) further indicated significant suppression of organic decomposition and gas formation in the ND‐ZS/ZF electrolyte system. The comprehensive performance radar chart (Figure 6E) clearly highlights the multiple benefits of adopting ND‐ZS/ZF electrolyte. After 1000 cycles, the pouch cell with ZS/ZF electrolyte showed a significant internal pressure rise to 2819.2 N, a 72.1% increase from its inactive state (1638.0 N), with evident localized stress concentration. In contrast, the ND‐ZS/ZF electrolyte cell exhibited a much lower internal pressure of 2030.7 N, only a 26.0% increase, coupled with more uniform pressure distribution (Figure 6F). This improvement is primarily attributed to the ND additive's ability to suppress gas evolution, water decomposition, corrosion reactions, and dendrite growth, thereby stabilizing zinc deposition and enhancing overall battery safety.

Thermal simulation analyses further indicated severe localized hotspots in ZS/ZF electrolyte pouch cells working after 1000 cycles, with peak postdischarge internal temperature exceeding 74 °C and steep temperature gradients. The ND‐ZS/ZF system exhibited a significantly lower maximum post‐discharge temperature of ≈67 °C with more uniform heat distribution (Figure S23, Supporting Information). It should be pointed out that the thermal simulations did not incorporate environmental cooling conditions, leading to substantially higher simulated temperatures compared to actual experimental measurements. Infrared thermal imaging of the pouch cells (Figure 6G) working under practical conditions (in a constant‐temperature laboratory at 25 °C cooled by air conditioning) confirmed this trend, i.e., the cells with ZS/ZF electrolyte experienced a noticeable postdischarge temperature rise of 6.2 °C from 29.1 °C (after 5 cycles) to 35.3 °C (after 1000 cycles), and under the same environmental conditions, the pouch cell with ND‐ZS/ZF electrolyte has only a minimal increase of 0.7 °C (from 29.8 to 30.6 °C). Further taking into account the influence of the pouch cell's tab design on the internal current and the corresponding temperature changes,^[^ ^53^, ^54^ ^]^ both simulations and experimental observations consistently demonstrate the effect of suppressing temperature rise by ND additive.

According to Newton's Law of Cooling (*Q*_cool_ = *hA*Δ*T*, where *Q*_cool_ in W is rate of heat dissipation, *h* in W m^−^^2^·K is heat transfer coefficient, *A* in m^2^ is surface area of the battery exposed to cooling), Δ*T* (*T*_bat_‐*T*_env_) in K is temperature difference between battery and environment), lower heat output Δ*T* directly reduces the cooling demand. The pouch cells with ND‐ZS/ZF electrolyte exhibit slower temperature rise compared to the cells with traditional ZS/ZF electrolyte, the system requires less active cooling (smaller Δ*T*), cutting thermal management energy costs. Based on the data in Figure 6G, when the *T*_env_ is 298 K, Δ*T* ZS/ZF and ND‐ZS/ZF cells after 1000 cycles are 10.3 and 5.6 K, respectively. This suggests that the heat dissipation requirement for the ND‐ZS/ZF pouch cell is nearly halved compared to the conventional system. Such thermal mitigation becomes especially critical under high‐rate cycling, where the ND‐containing electrolyte maintains milder temperature gradients. As a result, thermal management systems can be simplified, reducing associated energy consumption and operational costs in practical applications.

From an economic perspective, reducing thermal loads within AZIBs through the use of ND‐ZS/ZF electrolyte can significantly lower the energy consumption demands for cooling systems, thereby reducing overall operational energy consumption and associated costs. Moreover, lower temperature rise and reduced volume expansion help extend battery lifespan by mitigating thermal stress and associated performance deterioration. Considering the low‐cost and commercially feasible nature of ND additives, the ND‐ZS/ZF electrolyte system holds considerable practical potential, promising substantial economic and safety benefits for large‐scale energy storage applications.

## 3 Conclusion

In summary, this work introduces a novel electrolyte engineering strategy involving the incorporation of nanodiamonds (NDs) into a commercial electrolyte for aqueous zinc‐ion batteries (AZIBs), which effectively mitigates heat generation and internal pressure during cycling. The introduced NDs significantly enhance hydrogen‐bonding interactions within the electrolyte, facilitating a more uniform zinc‐ion distribution and reducing zinc‐ion desolvation energy. This mechanism considerably suppresses hydrogen evolution reactions and curtails the formation of corrosive by‐products, thereby substantially improving the corrosion resistance and interfacial stability of the zinc anode. Additionally, the enhanced thermal conductivity and specific heat capacity imparted by NDs significantly improve the electrolyte's heat dissipation capabilities, alleviating localized temperature increases caused by side reactions, especially under high current densities. Consequently, the diminished hydrogen evolution effectively limits the internal pressure build‐up in pouch cells. In practical terms, Zn||Zn symmetric cells employing ND‐ZS/ZF electrolyte demonstrate remarkable cycling stability, achieving up to 3300 h of stable operation at a high current density of 5 mA cm^−2^. Furthermore, Zn||VO_2_ pouch cells utilizing the ND‐ZS/ZF electrolyte retain more than 60% of their initial capacity after 1000 cycles. Comparative testing reveals that after 1000 cycles, pouch cells employing the conventional ZS/ZF electrolyte exhibit a post‐discharge temperature increase of 6.2 °C and a pressure increase of 72%. In contrast, the addition of ND to the ZS/ZF electrolyte significantly mitigates these effects, limiting the postdischarge temperature increase to only 0.7  °C and the pressure rise to 23.9%. Therefore, the ND‐ZS/ZF electrolyte comprehensively addresses the coupled challenges associated with zinc dendrite formation, hydrogen evolution, and thermal and pressure management through interface optimization and improved heat dissipation. This study provides valuable insights into developing high‐performance energy storage systems by introducing effective yet straightforward electrolyte additives.

## Conflict of Interest

The authors declare no conflict of interest.

## Supporting information



Supporting information

## Data Availability

The data that support the findings of this study are available from the corresponding author upon reasonable request.
