# Supplementary material for: Nanodiamond Regulated Electrolyte Enhances Thermal, Chemical and Structural Properties for Highly Reversible Zn Metal Anodes
Source: Adv Sci (Weinh). 2026 Jan 12;13(11):e16623. doi: 10.1002/advs.202516623 (PMC12931171; doi:10.1002/advs.202516623)
Supplement: Supplementary file 1 — Supporting information [file ADVS-13-e16623-s001.docx]

Supporting Information

**Nanodiamond Regulated Electrolyte Enhances Thermal, Chemical and Structural Properties for Highly Reversible Zn Metal Anodes**

Jiayan Zhu^1^, Xuan Gao^*2^, Nan Gao ^1^, Shaoheng Cheng ^1^, Junsong Liu^1^, Yuhang Dai ^3^*, Zhengxiao Guo*^2^, and Hongdong Li*^1^

^1^ State Key Laboratory of High Pressure and Superhard Materials, College of Physics, Jilin University, Changchun 130012, PR China

^2^ Department of Chemistry, The University of Hong Kong, Pokfulam Road, Hong Kong SAR, PR China

^3^ Thom Building, Department of Engineering Science, University of Oxford, Oxford, UK

**Email:** hdli@jlu.edu.cn; xuangao@hku.hk; zxguo@hku.hk; yuhang.dai@eng.ox.ac.uk

**Supplementary methods**

**Preparation of electrolyte and electrode**

ZS/ZF electrolyte was prepared by dissolving 1 M ZnSO_4_ (Macklin, >99.99%) and 1 M Zn(OTf)_2_ (Macklin, >99.99%) in deionized water. Nanodiamond (ND, 5–10 nm, Nanjing Mingchang New Material Technology Co., Ltd.) was added to the ZS/ZF electrolyte at concentrations of 0, 0.05, 0.1, and 0.5 wt%, followed by ultrasonication for 30 min to ensure uniform dispersion. Unless otherwise specified, 0.1 wt% ND-ZS/ZF was used for all electrochemical tests.

The VO_2_ cathode was prepared by mixing VO_2_, Super P conductive carbon black, and polyvinylidene fluoride (PVDF) at a weight ratio of 8:1:1 in N-methyl-2-pyrrolidone (NMP). The resulting slurry was uniformly coated onto 30 μm stainless steel foil, followed by drying at 60 °C overnight. The active material loading was controlled at 6-7 mg cm^-2^. All materials were obtained from Zhejiang Vastech Co., Ltd.

**Battery fabrication**

CR2032-type coin cells were assembled. Glass fiber membrane (GF/A, 290 μm, Chongqing Ouleji Co., Ltd) is used as separators (18 mm). The zinc anode is a 16 mm diameter disc cut from commercial zinc foil (50 μm, Zhejiang Vastech Co., Ltd.), and the cathode (180~200 μm) is also a 16 mm diameter disc. Each cell was assembled with 180 μL of the prepared electrolyte.

For pouch cells, VO_2_ was used as the cathode and zinc foil as the anode, both cut into 15 × 13 cm^2^ rectangles. Glass fiber membrane was used as the separator. Three cathode layers were stacked and sealed in an aluminum-laminated pouch. Each pouch cell was filled with 328 mL of the prepared electrolyte. The structure of the pouch cell is shown in Figure S21.

All ND-modified electrolytes for coin and pouch cells assembly were prepared in a single batch, thoroughly mixed, and stored in airtight containers to prevent moisture absorption and sedimentation. Before use, the electrolyte was ultrasonicated and filtered to ensure uniform nanodiamond dispersion and remove any aggregates.

For coin and pouch cells, zinc foil was cut from the same metal roll, and electrode fabrication, including cathode slurry preparation and coating, used single batches to maintain uniformity. The nanodiamond content in the electrolyte was precisely controlled by mass ratio, and all cells within each group used electrolyte from the same preparation. Prior to injection, the electrolyte was pre-degassed to reduce air bubble formation.

Following assembly and electrolyte injection, pouch cells were rested for six hours at room temperature (25 ℃) to ensure complete wetting and interface stabilization. The structure and material sources for all pouch cells remained consistent as described above.

**Electrochemical measurements**

Electrochemical tests were performed using a Neware battery test system (CT-4008 and CT-4016 5 V 30A) at room temperature (25 ℃). Coin and pouch cells underwent galvanostatic cycling, rate capability, and long-term cycling tests within a voltage window of 0.3-1.7 V (vs. Zn/Zn^2+^). Zn||Zn symmetric and Zn||Cu asymmetric coin cells were assessed for plating/stripping stability and coulombic efficiency, respectively. Cyclic voltammetry (CV), chronoamperometry (CA), Tafel, linear sweep voltammetry (LSV), and electrochemical impedance spectroscopy (EIS) were conducted on an electrochemical workstation (CHI660E). Thermal behaviors during cycling were monitored by infrared imaging.

**Material Characterizations**

The morphology and structure of the detonation NDs were analyzed using transmission electron microscopy (TEM, JEM-2200FS, JEOL) and scanning electron microscopy (SEM, S-4800, Hitachi Limited). The elemental distribution of the samples was determined by energy-dispersive X-ray spectroscopy (EDS, Oxford Instruments). The crystal structure of the nanodiamonds was analyzed using X-ray diffraction (XRD, Rigaku D/MAX-RA). The pH and conductivity of the electrolyte were measured using a pH meter and conductivity meter (Shanghai Leimagnetic). The Fourier-transform infrared spectroscopy (FTIR) (Thermo Scientific Nicolet iS50) was used to measure the absorption spectra of electrolytes. The contact angle of the electrolyte on the electrode surface was measured using a contact angle goniometer (Jiangnan JC2000D) to evaluate its wettability. In-situ differential electrochemical mass spectrometry (DEMS) were performed using the PM-DEMS system from Shanghai Jingpu Ruo Technology Co., Ltd. A thermal imaging camera (FLIR E6, FLIR Systems) was used to monitor the temperature variation of the batteries in real time during charge-discharge cycling. The specific heat capacity of the samples was measured by differential scanning calorimetry (DSC, Sapphire method, TA DSC25). The thermal conductivity of the ZS/ZF and ND-ZS/ZF electrolytes was measured using the Hot Disk method (TPS2500S, Hot Disk AB) at 25 ℃. External compression conditions of the soft-pack battery were tested using a distributed thin-film testing system (DPM-8U, TianCe Technology).

**Calculation Setup**

The ionic conductivity ($\sigma$, S m^-1^) of the electrolytes were calculated by the equation (Equation S1) according to EIS tests with a frequency range from 0.01 Hz to 1 × 10^5^ Hz.

$$\sigma=\frac{L}{R\times A} (Eqution S1)$$

where $L$, $A$, and $R$ are the thickness (0.058 cm), electrode contact area (2.01 cm^2^), and bulk resistance, respectively.

The specific heat capacity (*C_p_*​) of the electrolytes was determined based on the fundamental thermodynamic relationship:

$$C_{p}=\frac{q}{m\cdot\Delta T} (Equation S2)$$

where $q$ is the amount of heat absorbed (J), $m$ is the mass of the electrolyte sample (g), $C_{P}$​ is the specific heat capacity (J g^-1^ K^-1^), and $\Delta T$ is the temperature change (℃).

The thermal diffusivity ($\alpha$) of the electrolyte was calculated using Equation S3:

$$\alpha=\frac{k}{\rho C_{P}} (Equation S3)$$

where $k$ is the thermal conductivity (W m^-1^ k^-1^), $\rho$ is the density (g cm^-3^), and $C_{P}$​ is the specific heat capacity (J g^-1^ K^-1^). This relationship describes how efficiently heat propagates through the material.

Corrosion current density ($I_{corr}$) is an important electrochemical parameter for evaluating the corrosion rate of a material, defined as the current density passing through a unit area of the material during the corrosion process.

$$I_{corr}= \frac{m_{Zn}}{t} (Eqution S4)$$

where $m_{Zn}$ is the total mass of deposited Zn metal and $t$ is the corrosion time corresponding to the complete consumption of Zn.

The relationship between the critical Zn nucleus radius ($r_{crit}$) and the NOP ($\eta$) is by Equation S5*:*

$$r_{crit}=2\frac{\gamma V_{m}}{F|\eta|} (Eqution S5)$$

where $\gamma$ represents the surface energy at the electrode-electrolyte interface; $V_{m}$ is the Zn molar volume.

The Zn||Zn cells were subjected to constant potential (20 mV) for 120 min, and the impedance before and after the polarization was recorded. The $t_{{Zn}^{2+}}$was determined by the following equation Equation S6:

$$t_{{Zn}^{2+}}= \frac{I_{SS} (\Delta V-R_{0}I_{0})}{I_{0} (\Delta V-R_{SS}I_{SS})} (Eqution S6)$$

where $t_{{Zn}^{2+}}$ is Zn^2+^ transference number, $I_{0}$ and $I_{SS}$ are the initial and steady current, $R_{0}$ and $R_{SS}$ the initial and steady resistance, and $\Delta V$ the applied voltage (20 mV).

Binding energy (BE) is calculated using first-principles density functional theory (DFT). The computational software used is VASP (Vienna Ab-initio Simulation Package), based on plane-wave basis sets and pseudopotential methods. The calculation is performed using the following formula:

$${BE=E}_{\mathrm{total}}-E_{\mathrm{isolated}}-E_{\mathrm{substrate}} (Eqution S7)$$

where *BE* is binding energy, $E_{\mathrm{total}}$ is the total energy of the adsorbed system; $E_{\mathrm{isolated}}$ is the isolated energy of the adsorbate; $E_{\mathrm{substrate}}$ is the isolated energy of the substrate material.

The diffusion coefficient (D) related to the particle’s radius and mediumv iscosit is based on Stokes-Einstein equation (Equation S8).

$$D=\frac{k_{B}T}{6\pi\eta r} ( Eqution S8)$$

where $D$ is diffusion coefficient, $\eta$ is viscosity of the solution, $T$ is temperature, $r$ is radius, $k_{B}$ is the Boltzmann constant. This relationship highlights that as the temperature increases, the diffusion coefficient of zinc ions also increases, thereby enhancing their mobility.

The de-solvation energy ($E_{a}$) was determined by measuring the impedance variation under different temperatures (0 - 50 ℃) and then by fitting the data according to the Arrhenius equation Equation S9$:$

$$\frac{1}{R_{s}}=A\exp\left( -\frac{E_{a}}{RT} \right) (Eqution S9)$$

where $R_{s}$ is the interfacial impedance, $A$ is the pre-exponential factor, $R$ is the universal gas constant (8.314 J K^-1^ mol^-1^) and $T$ is the absolute temperature.

The exchange current density ($i_{0}$) is the current density at the electrode surface when the forward and reverse reaction rates are equal at equilibrium. It reflects the rate of charge exchange between the electrode and the solution, determining the electrochemical activity of the electrode.

$$i=i_{0}\frac{F}{RT}\frac{\eta}{2} (Eqution S10)$$

where $i$ is the current density, $i_{0}$ is the exchange current density, $\eta$ is the total overpotential, $F$ is the Faraday constant (96485.33 C mol^-1^), $R$ is the gas constant (8.314 J K^-1^ mol^-1^), and $T$ is the absolute temperature (298K).

In full batteries, Equation S10 is used to describe the relationship between the current and voltage, particularly during the charge and discharge processes, to analyze the electrochemical dynamics of the battery.

$$i\left( v \right)=av^{b} ,\log\left( i \right)=\log\left( a \right)+b\times\log\left( v \right) (Eqution S11)$$

where the slope part ($b$) indicates the type of electrochemical reaction. The $b$ value approaching 0.5 means that the electrochemical reaction is controlled by ionic diffusion, while the $b$ value close to 1 means the predominance of surface pseudocapacitance reaction during the electrochemical process.

**Theoretical calculations**

All MD simulations were performed using the GAFF2 force field. The ACPYPE was employed to obtain the GAFF2 force field topology. The simulation box size was 9 × 9 × 9 nm^3^ for all simulation models, which consisted of Zn^2+^, SO_4_^2-^, OTF^-^, H_2_O, with/without ND molecules. The cut-off distance of 1.2 nm was used for a Lennard–Jones potential. The Coulombic potential was calculated using particle mesh Ewald (PME) with a cut-off distance of 1.2 nm and Fourier grid spacing of 0.12. All bonds were constrained with the LINCS algorithm. and periodic boundary conditions were applied in all directions. The MD simulations were started by running initial energy minimisation, followed by 500 ps of NVT simulation and 500 ns of NPT simulation, with an integration time step of 0.001 ps. All simulation systems were finally maintained at 298 K using the Nose–Hoover thermostat for 70 ns to collect simulation data. A time constant of 1 ps was applied for the temperature coupling. Several Zn-ion structures observed from the MD simulations were then taken for further investigation using density functional theory (DFT). The DFT calculations were implemented using the Vienna abinitio simulation package (VASP) with the core and valence electronic interactions being modelled using the projector augmented wave (PAW) method.^[1]^ The Perdew–Burke–Ernzerhof (PBE) exchange-correlation function was employed. The wavefunction was expanded with a kinetic energy cut-off of 500 eV and Gamma k-points were used. The dispersion correction was also considered in this study by using the DFT-D3 method.^[2]^ To investigate the thermal performance of pouch cells, we conducted thermal simulation analyses based on the structural and material parameters of real batteries. A three-dimensional thermal simulation model of the pouch cell was established, encompassing electrodes, separators, electrolytes, and casing, combined with experimentally measured electrochemical parameters (e.g., current density and heat generation rate). By Finite Volume Method (FVM),^[3,4]^ the temperature distribution and heat transfer behavior of the battery under various charge-discharge cycles and environmental conditions were simulated.


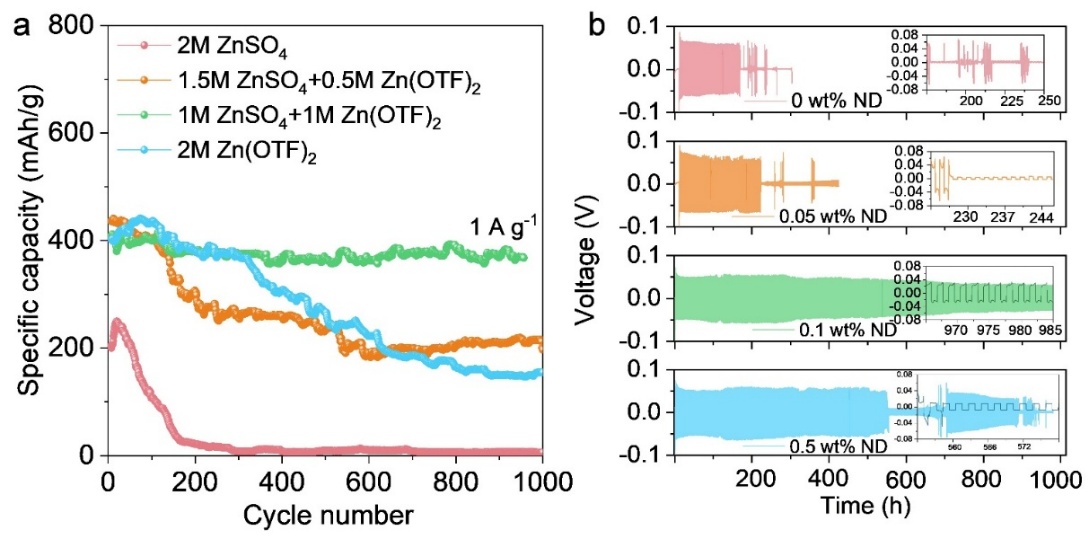


**Figure S1.** Electrolyte Screening. a) Long-term cycle performance of Zn||VO_2_ coin cell under different electrolytes at 1 A g^-1^. b) Long-term cycle performance of Zn||Zn symmetric cells in electrolytes with different amounts of NDs additions at a current density of 1 mA cm^-2^ with a capacity of 1 mAh cm^-2^.


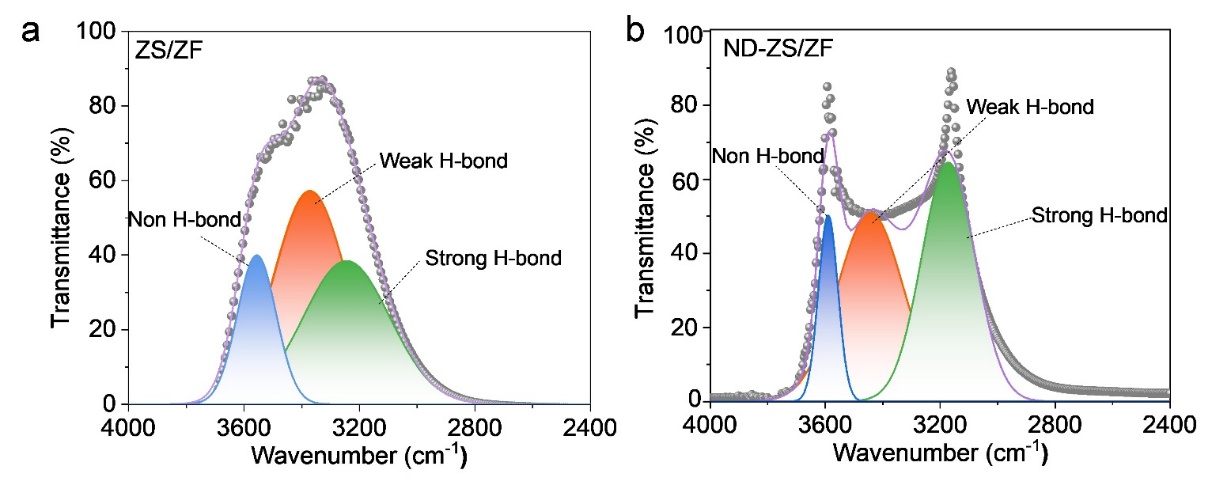


**Figure S2.** FTIR analysis of hydrogen bonding in different electrolytes. a) FTIR spectra and corresponding fitting curves in the range of 2400-4000 cm^-1^ for ZS/ZF electrolyte. b) FTIR spectra and fitting results for ND-ZS/ZF electrolyte.


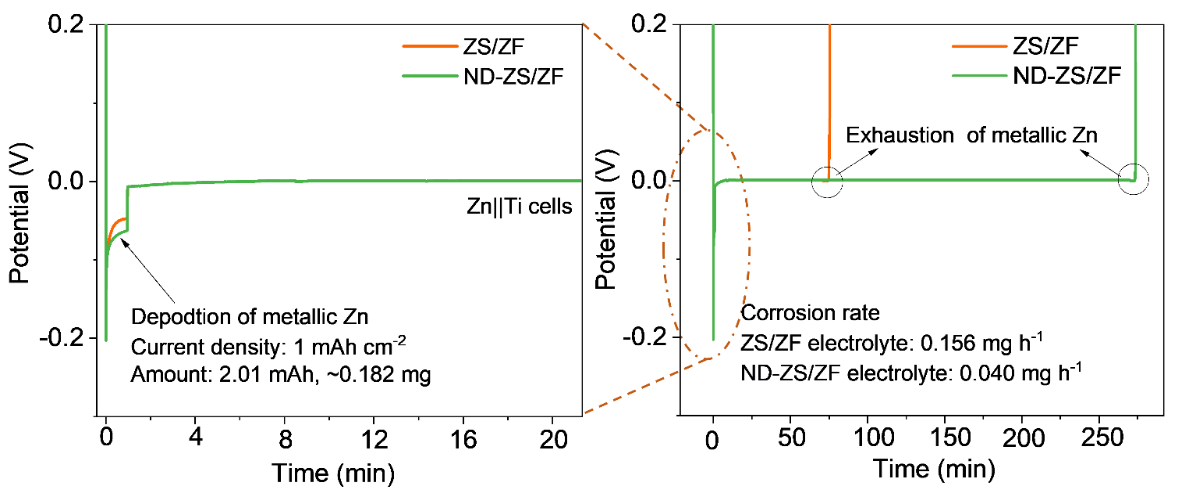


**Figure S3.** Corrosion rate of Zn anodes in two electrolyte. Voltage-time curves for Zn||Ti electrodes immersed in the ZS/ZF and ND-ZS/ZF electrolyte. Profiles were recorded for the initial 20 minutes and 300 minutes.


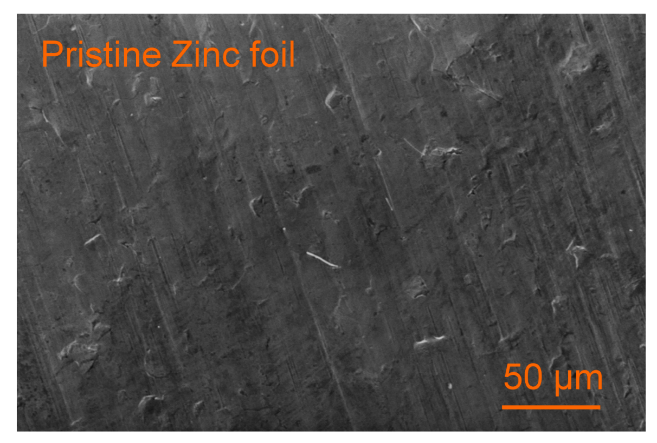


**Figure S4.** SEM image of pristine zinc foil.


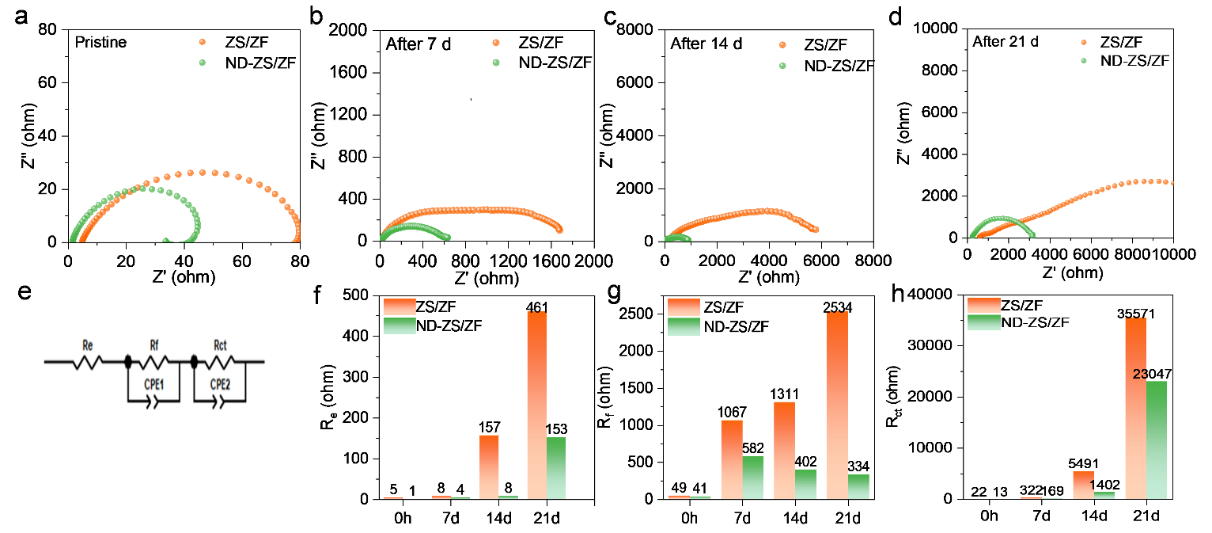


**Figure S5.** Interfacial impedance evolution of Zn||Zn symmetric cells stored in ZS/ZF and ND-ZS/ZF electrolytes. a–d) Nyquist plots of symmetric cells after different immersion durations: 0 h (a), 7 days (b), 14 days (c), and 21 days (d). e) Equivalent circuit model used for impedance fitting. f) Extracted total interfacial resistance (R_e_) during storage. g) Extracted film resistance (R_f_) during storage. h) Extracted charge transfer resistance (R_ct_) during storage.


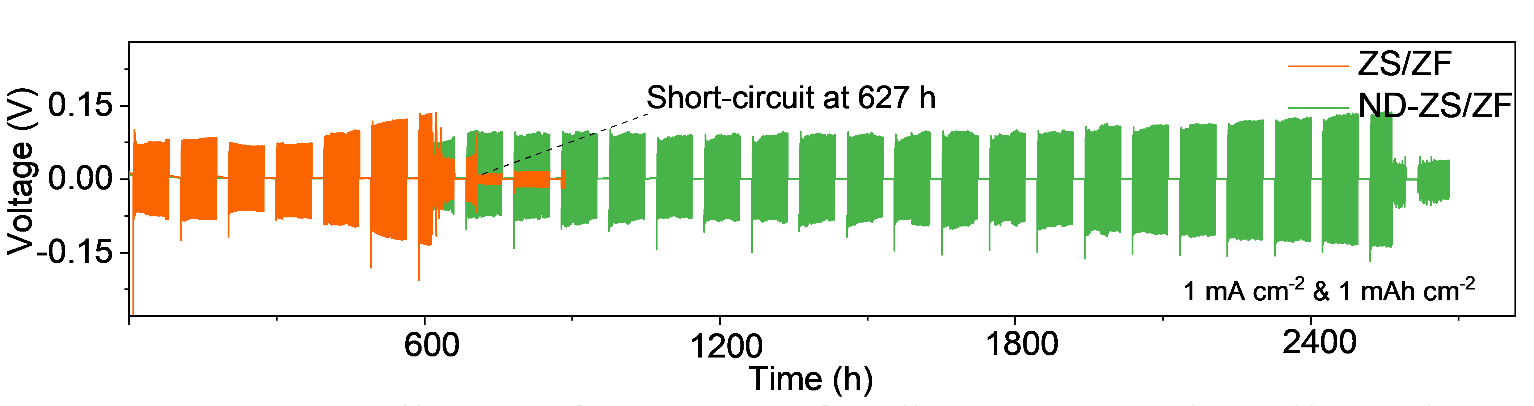


**Figure S6.** Cycling performance of Zn||Zn symmetric cells under intermittent galvanostatic charge/discharge testing at a current density of 1 mA cm^-2^, with a capacity of 1 mAh cm^-2^.


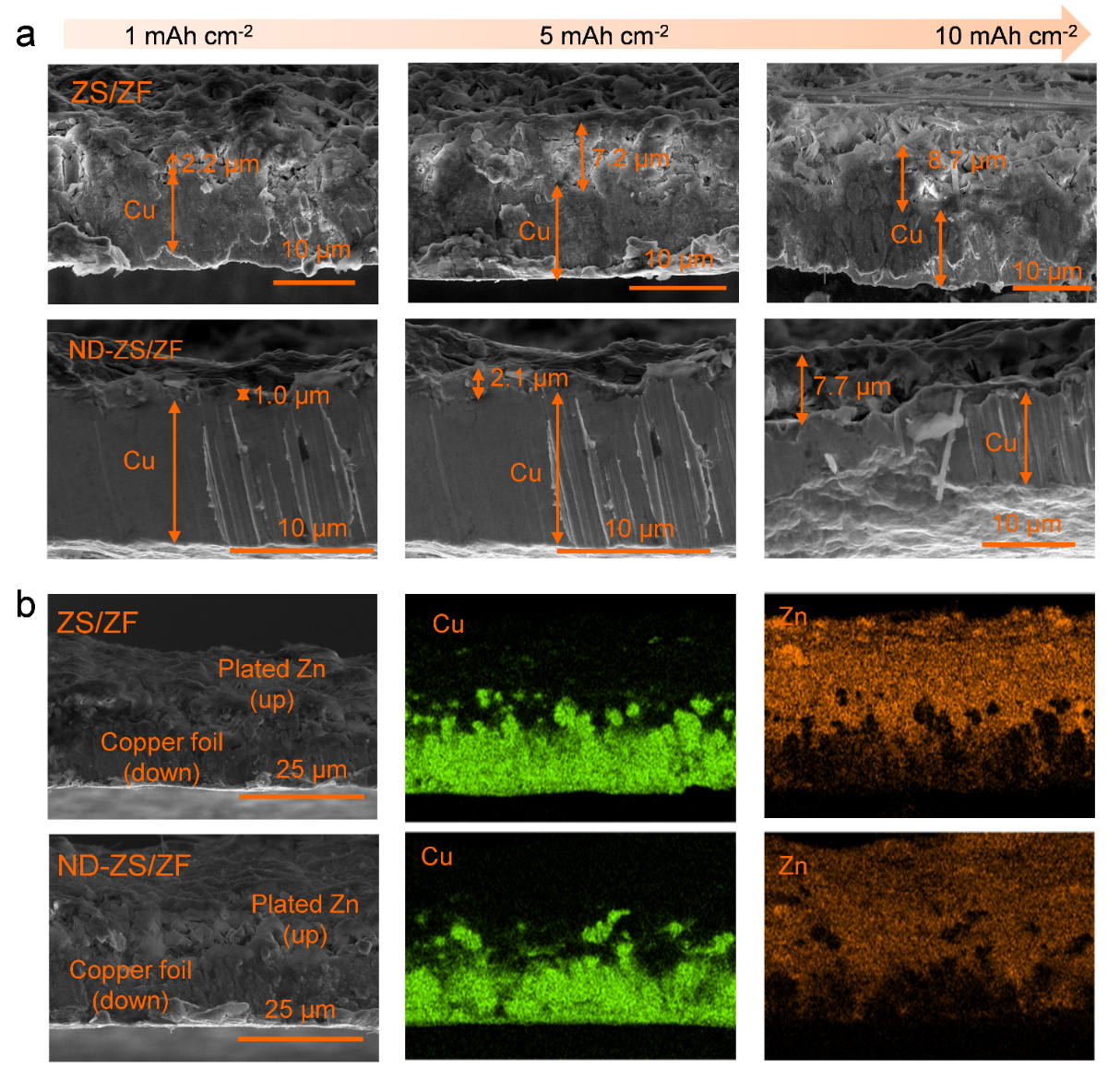


**Figure S7.** Cross-sectional morphology of Zn deposited on Cu current collectors. a) SEM images of Zn plating on Cu foil at 1 mA cm^-2^ with different areal capacities in ZS/ZF and ND-ZS/ZF electrolytes. b) Cross-sectional SEM comparison of Zn layers deposited from ZS/ZF and ND-ZS/ZF electrolytes.


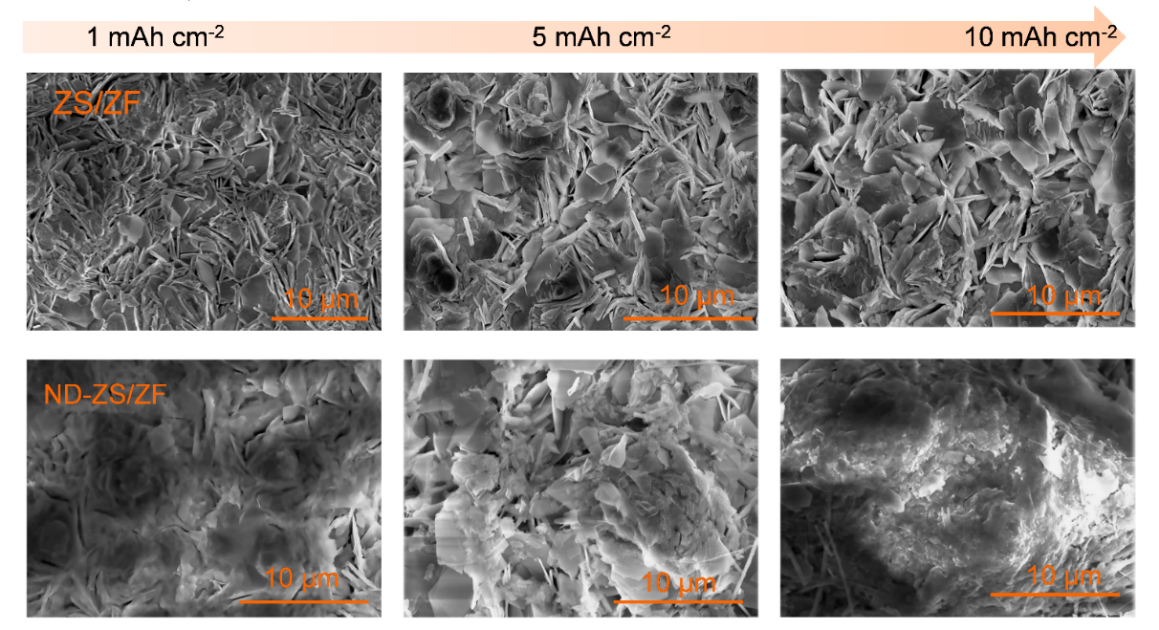


**Figure S8.** Planar morphology of Zn deposited on Cu current collectors at 1 mA cm^-2^ with varying areal capacities in ZS/ZF and ND-ZS/ZF electrolytes.


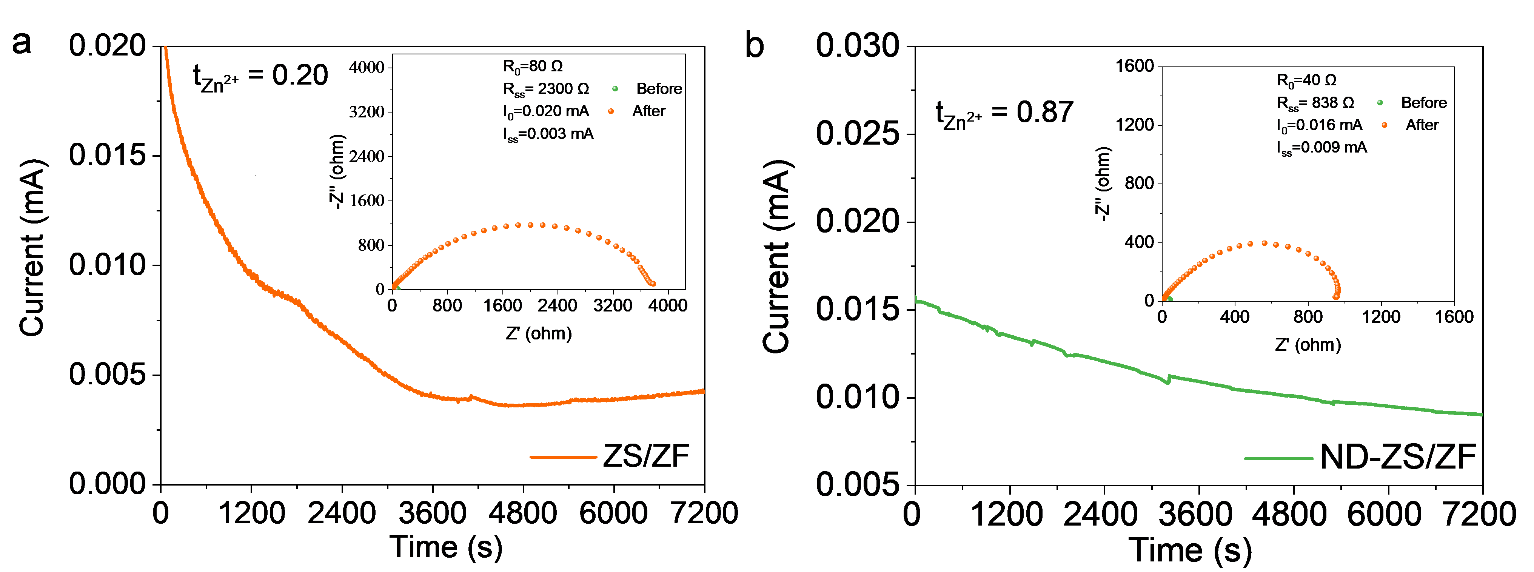


**Figure S9.** Transference number of Zn^2+^. Polarisation profile and Nyquist plots before and after polarization of Zn||Zn symmetric cells with (a) ZS/ZF and (b) ND-ZS/ZF.


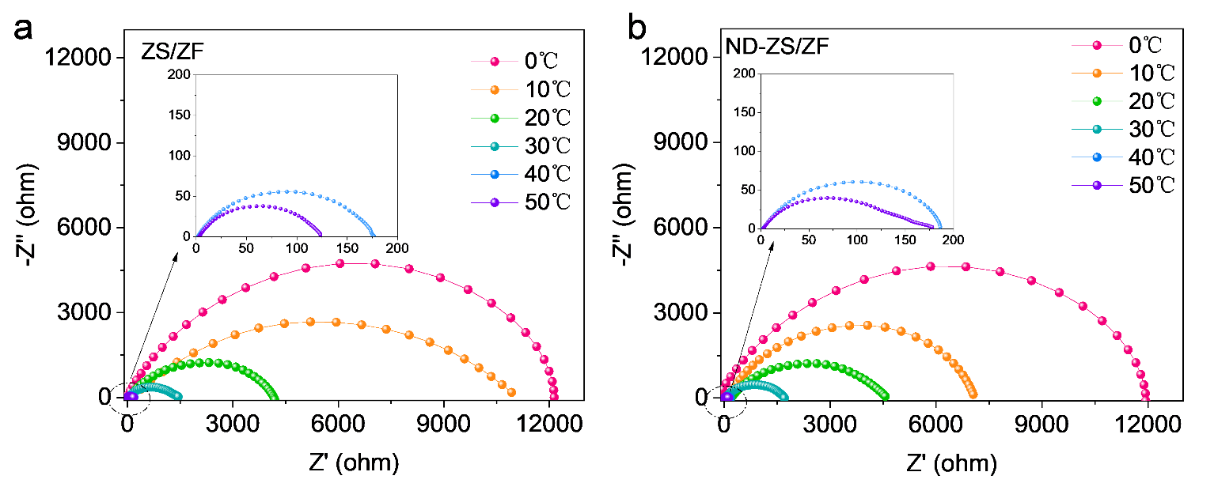


**Figure S10.** Nyquist plots of Zn||Zn symmetric cells testing under various temperatures in (a) ZS/ZF and (b) ND-ZS/ZF.


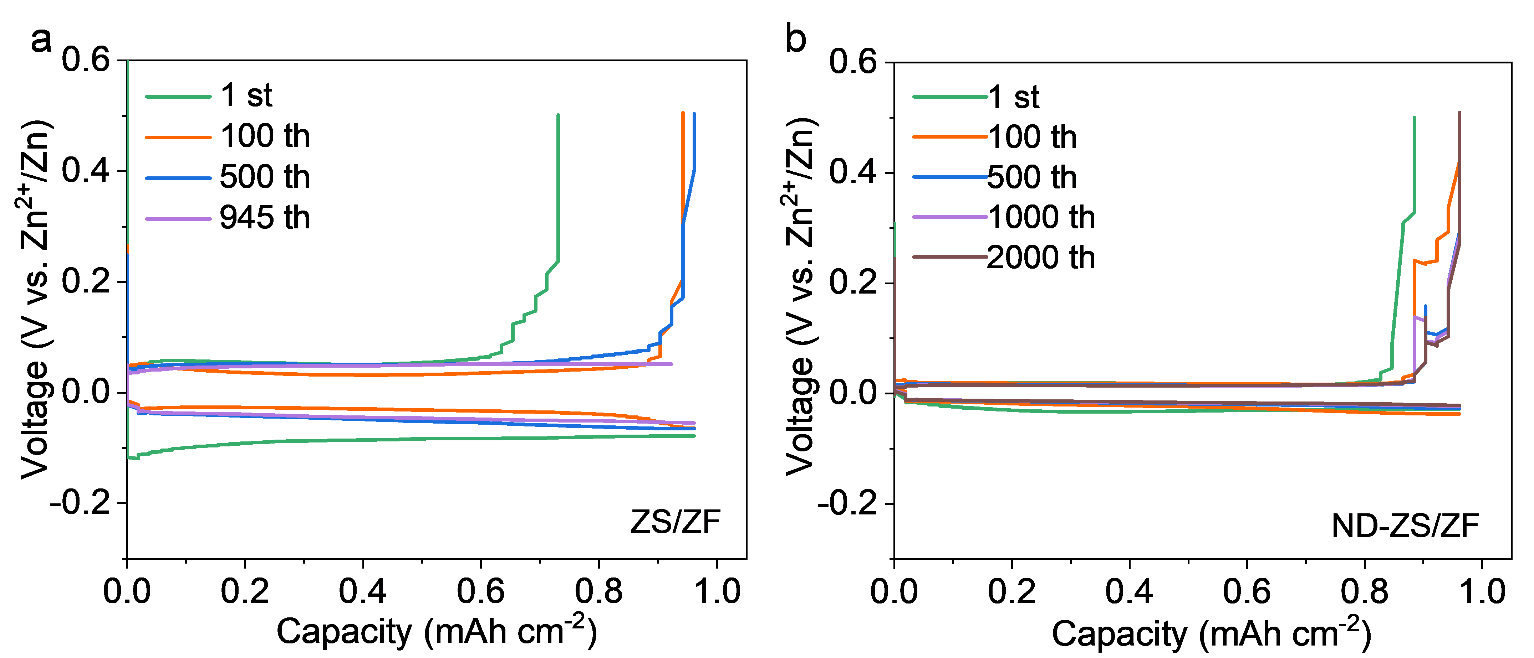


**Figure S11.** Voltage–capacity curves of Zn||Cu cells during cycling in (a) ZS/ZF and (b) ND-ZS/ZF electrolytes.


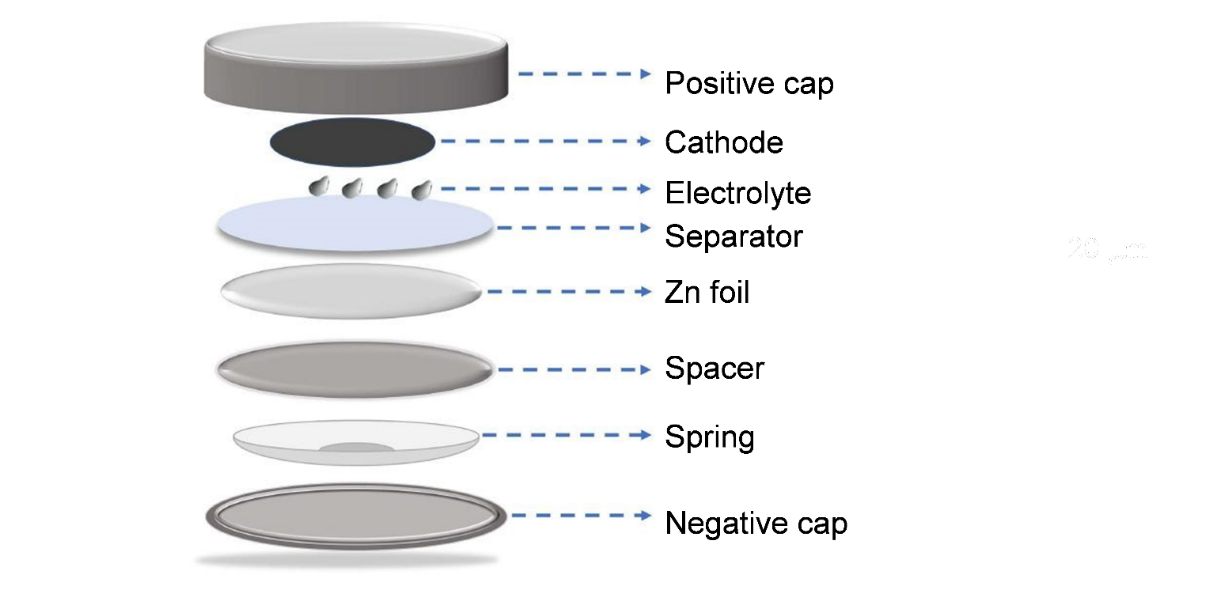


**Figure S12.** Schematic diagram of Zn-based coin cells using the electrolyte.


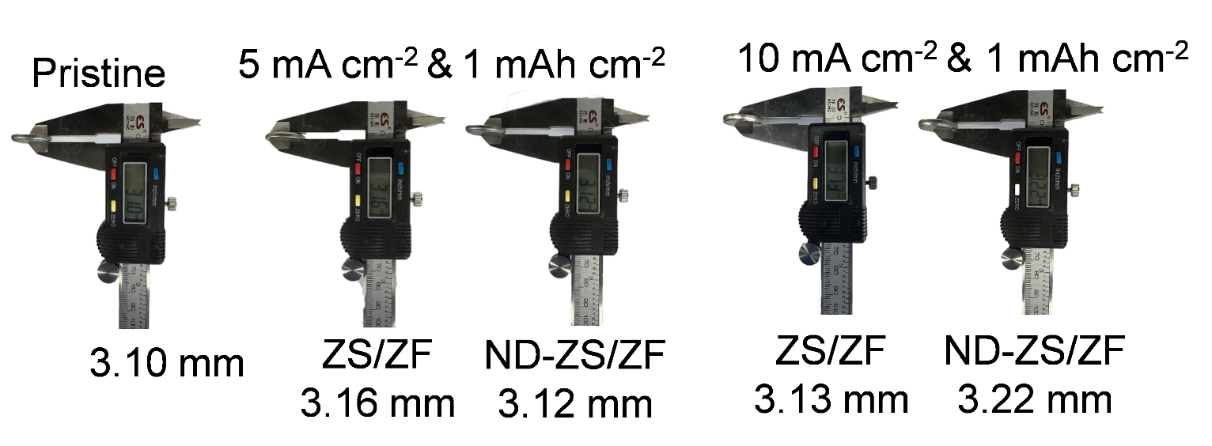


**Figure S13.** Thickness variation of coin cells after 100 cycles.


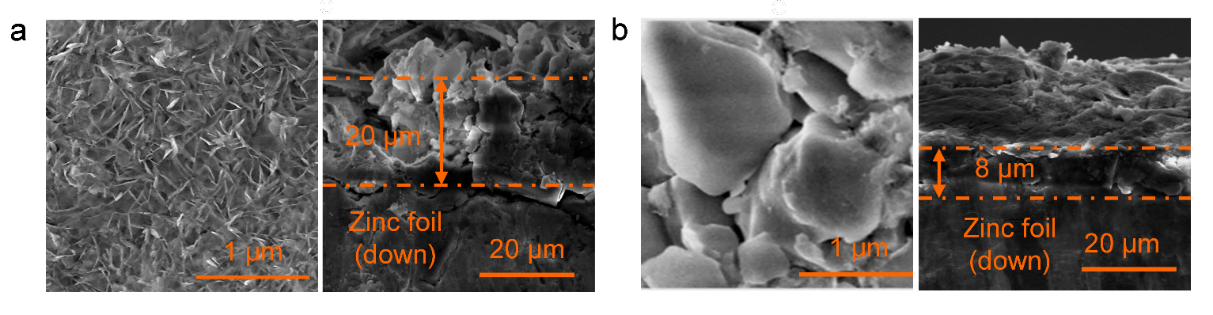


**Figure S14.** The show top and cross-sectional SEM images of zinc foil in (a) ZS/ZF and (b)ND-ZS/ZF electrolytes.


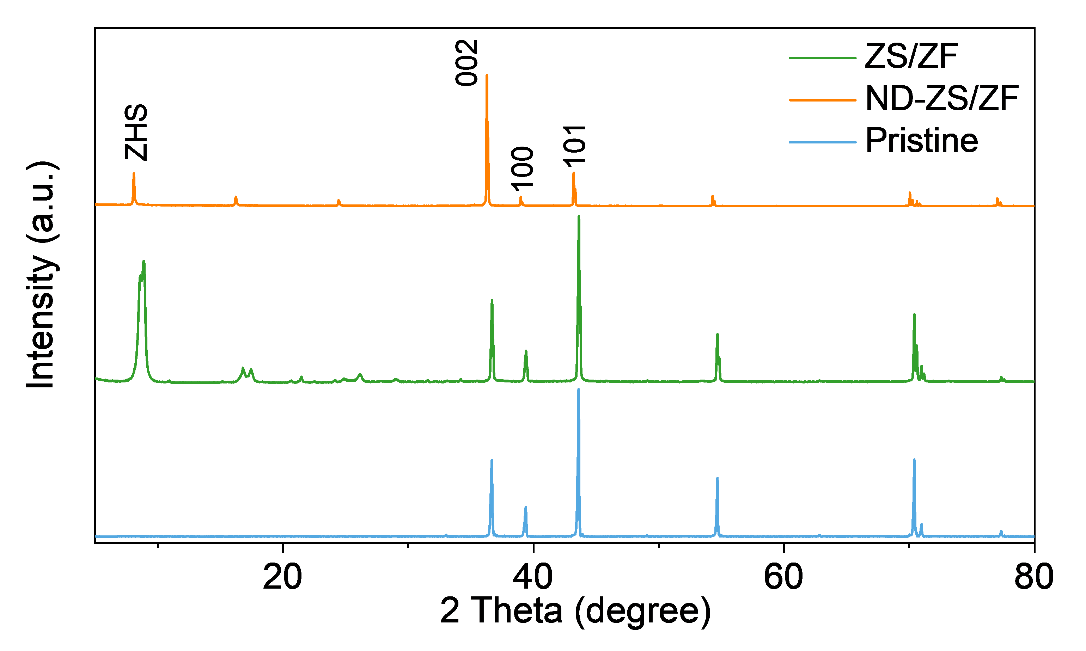


**Figure S15.** XRD patterns of zinc anode after 100 cycles in the Zn||Zn cells with ZS/ZF and ND-ZS/ZF electrolytes, with prinstine zinc anode for comparison.


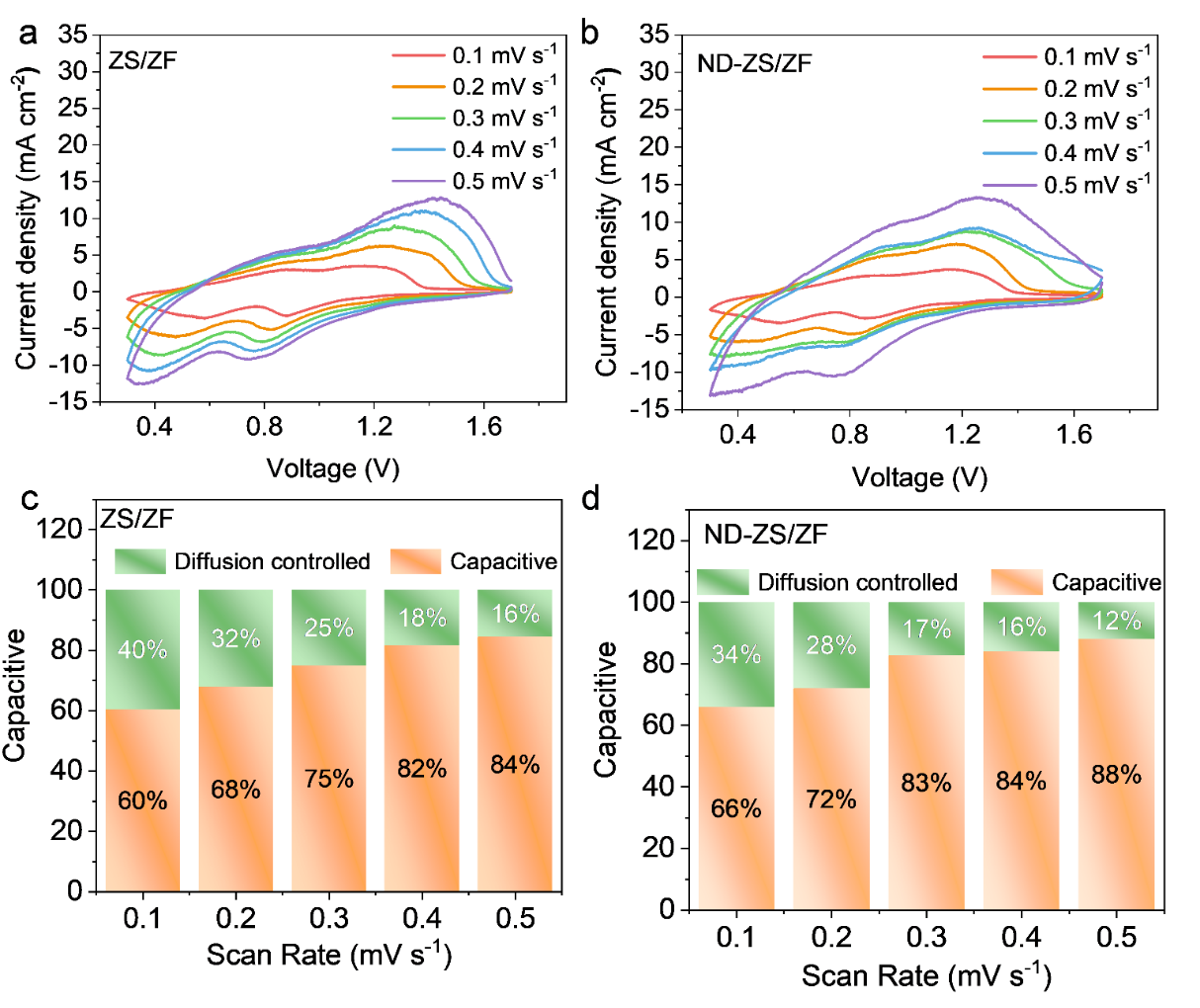


**Figure S16.** Capacitive behavior analysis of Zn||VO_2_ coin cells. a,b) CV curves at various scan rates in ZS/ZF and ND-ZS/ZF electrolytes. c,d) Separation of capacitive and diffusion-controlled contributions.


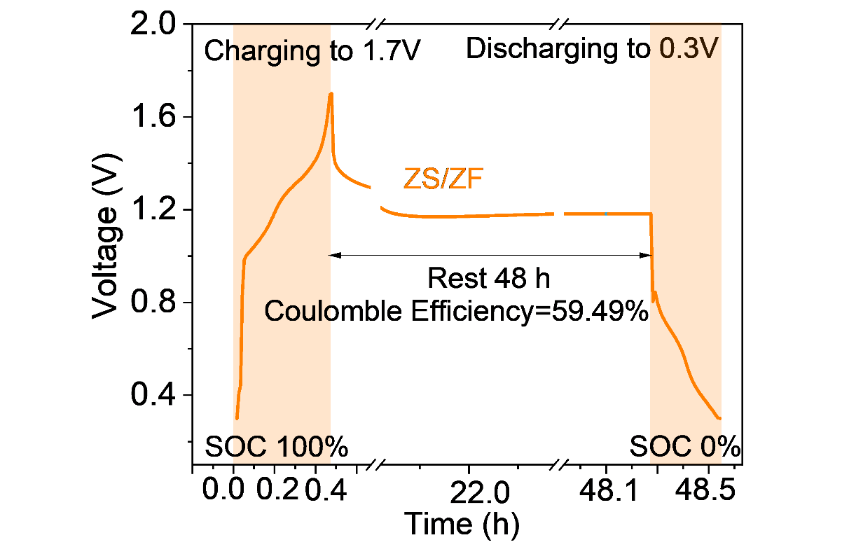


**Figure S17.** Self-discharge behavior of Zn||VO_2_ coin cells with ZS/ZF electrolyte after 48 h rest.


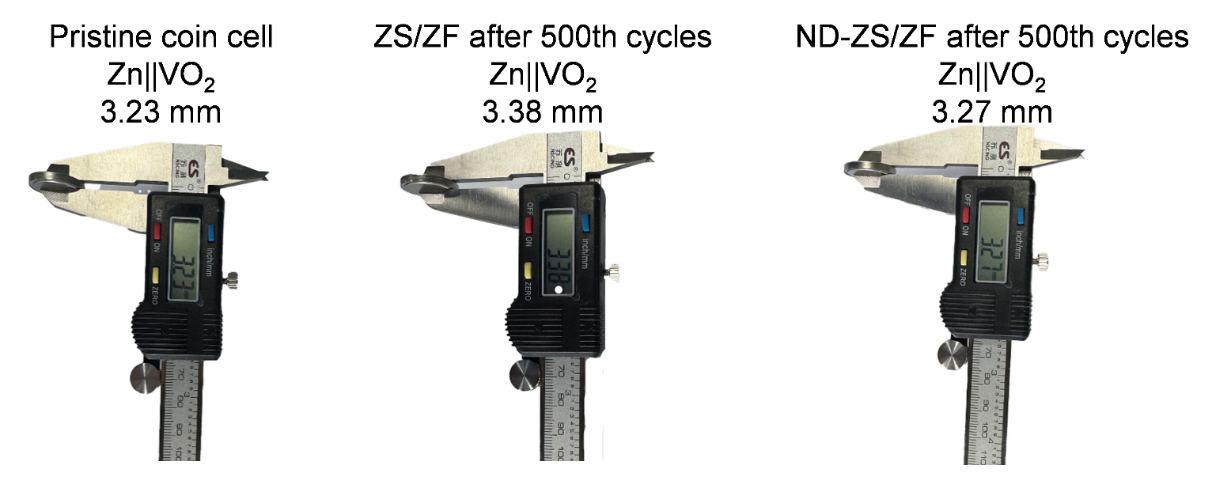


**Figure S18.** The thickness change of Zn||VO_2_ coin cell after 500 cycles.


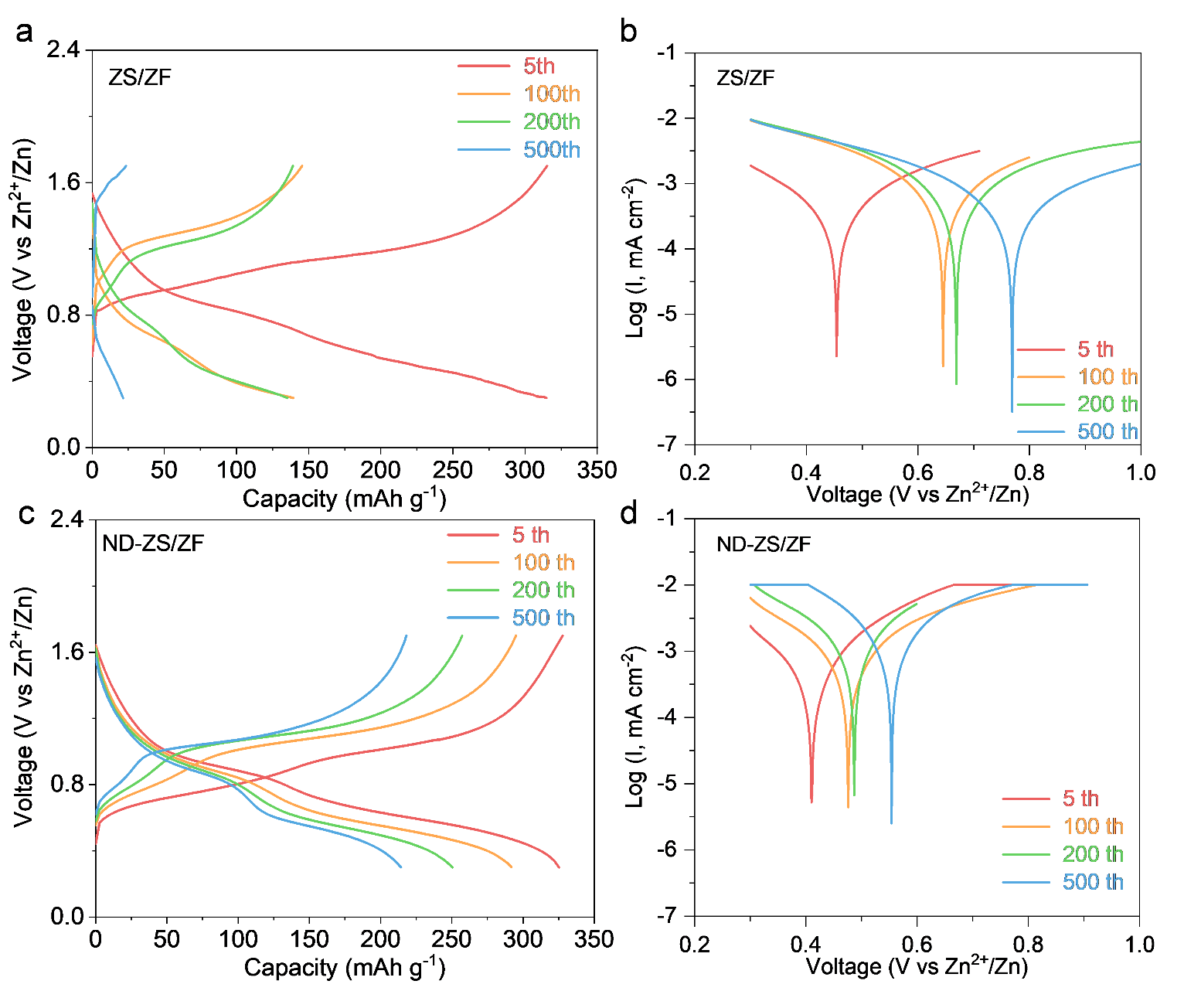


**Figure S19.** Polarization and corrosion behavior of Zn||VO_2_ cells. a,c) Charge–discharge curves at different cycles in ZS/ZF and ND-ZS/ZF. b,d) Corresponding Tafel plots reflecting corrosion current density evolution.


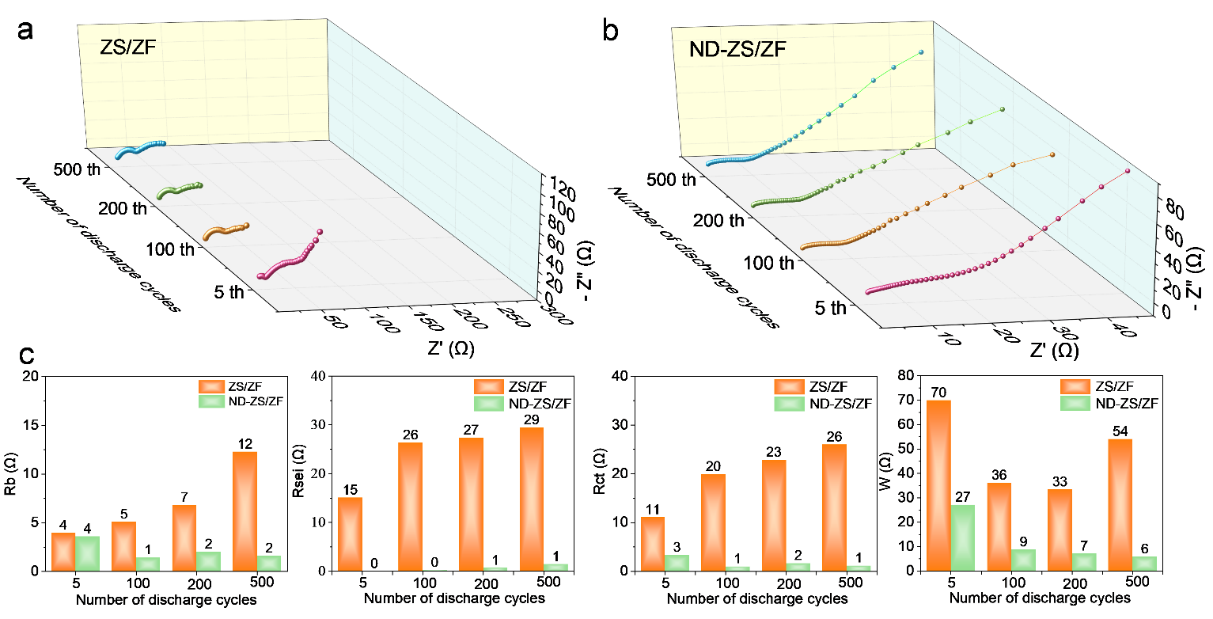


**Figure S20.** Electrochemical impedance characteristics of Zn||VO_2_ cells. a,b) Nyquist plots of ZS/ZF and ND-ZS/ZF cells at different cycles. c) Comparison of resistance components (R_b_, R_sei_, R_ct_, W) over 500 cycles.


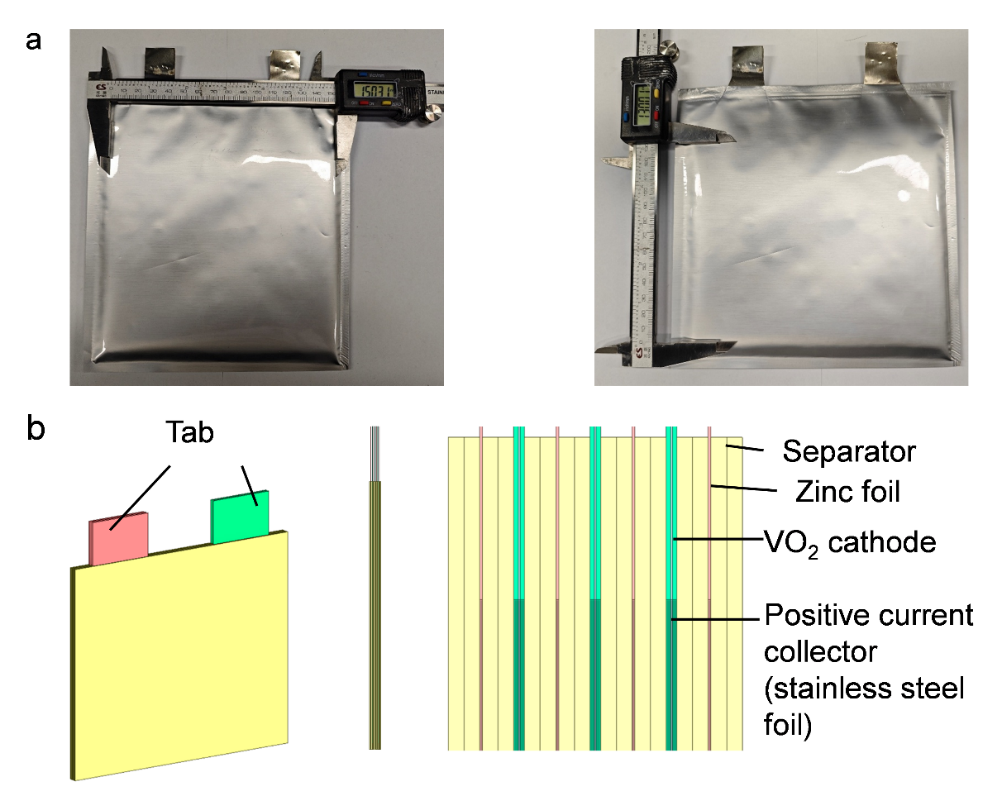


**Figure S21.** Structural design and dimensions of the assembled Zn||VO_2_ pouch cell. a) Assembled pouch cell with measured dimensions. b) Schematic of internal configuration.


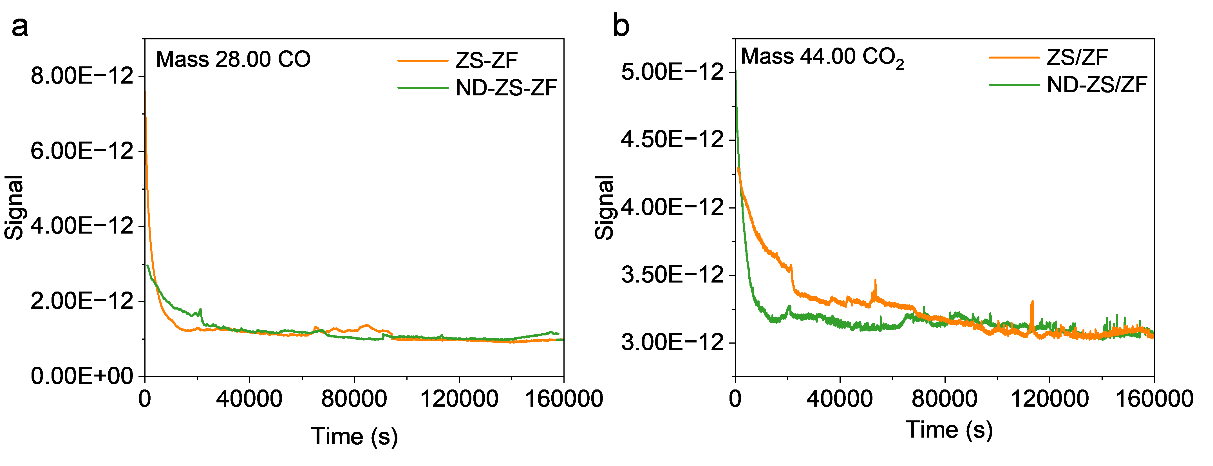


**Figure S22.** DEMS analysis of gas evolution in Zn||VO_2_ pouch cells with different electrolytes. a) CO signal profiles during cycling. b) CO₂ signal profiles during cycling.


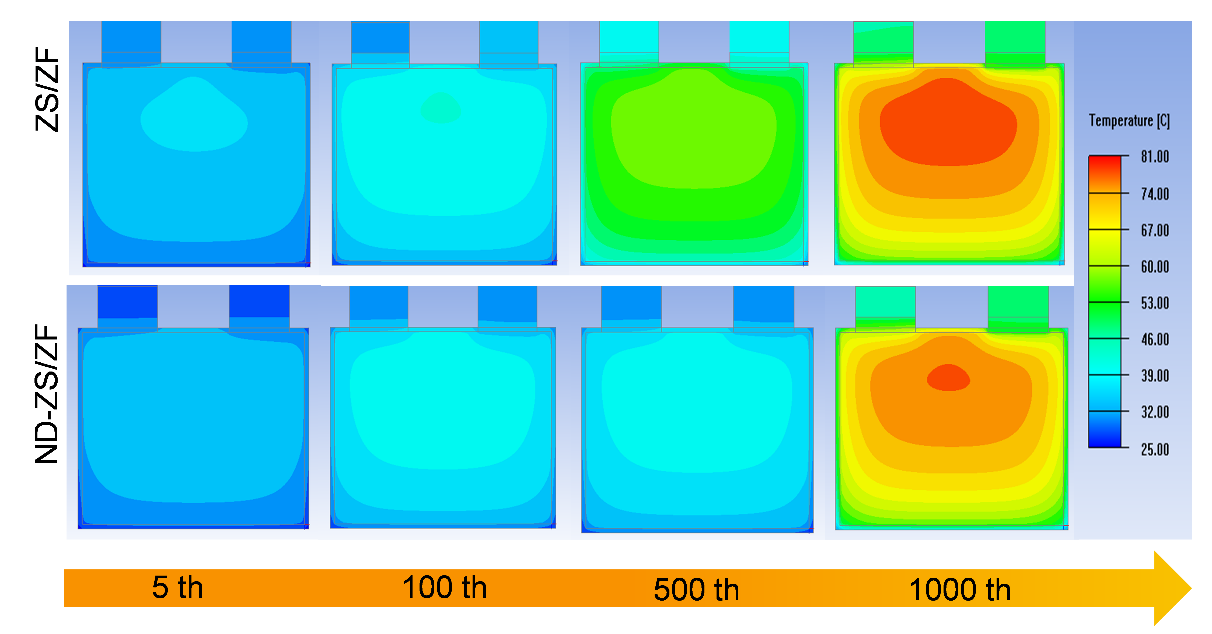


**Figure S23.** Thermal simulation calculations of the Zn||VO_2_ pouch cell during discharge at different cycle counts.

**Table S1.** Ion Conductivity of NDs compared to revious reported in aqueous electrolytes.

| Electrolytes | Salt | Solvent | Ionic conducticity (mS cm^-1^) | Ref. |
| --- | --- | --- | --- | --- |
| NDs | 1M ZnSO_4_ + 1M Zn(OTF)_2_ | H_2_O | 54.8 | This work |
| Zn(OTF)_2_ | 3M Zn(OTF)_2_ | H_2_O | 32.3 | ^[5]^ |
| 4-methyl-4-glycidylmorpholin bis | 2M ZnSO_4_ | H_2_O | 18.9 | ^[6]^ |
| Diacrylate-carboxymethyl cellulose | 2M ZnSO_4_ | H_2_O | 30.2 | ^[7]^ |
| Polyacrylamide | 3M ZnSO_4_ | H_2_O | 13.9 | ^[8]^ |

**Table S2.** Thermal conductivity and specific heat capacity of the material at 25 ℃.

| Sample Name | Thermal Conductivity  (W m^-1^ K^-1^) | Thermal Diffusivity  (mm^2^ s^-1^) | Specific Heat Capacity  (J (g ℃)^-1^) |
| --- | --- | --- | --- |
| H_2_O | 0.6060 | 0.1433 | 4.1823 |
| ND | 5.2353 | 2.5374 | 2.0134 |
| ZS/ZF | 0.5207 | 0.1495 | 2.7485 |
| ND-ZS/ZF | 0.5252 | 0.1682 | 2.8116 |

**Table S3.** Electrochemical stability windows of different electrolytes.

| Electrolytes | ESW (V) | Cathode | Ref. | Electrolytes |
| --- | --- | --- | --- | --- |
| ND-ZS/ZF | 1.83 | Zn | This work | ND-ZS/ZF |
| ZS/ZF | 1.57 | Zn | This work | ZS/ZF |
| Zn/K PAMs | 1.60 | ZnHCF | ^[9]^ | Zn/K PAMs |
| ZnSO_4_ | 1.16 | Zn | ^[10]^ | ZnSO_4_ |
| ZnSO_4_ | 1.27 | Zn@chitin | ^[10]^ | ZnSO_4_ |

**Table S4.** Summary of Nyquist-fit resistance elements (R_b_, R_sei_, R_ct_) and Warburg coefficient (W) for Zn||VO_2_ cells across 5^th^–500^th^ cycles.

| Cycle | Electrolyte | Rb (Ω) | Rsei (Ω) | Rct (Ω) | W (Ω) |
| --- | --- | --- | --- | --- | --- |
| 100 | ZS/ZF | 3.96 | 15.08 | 11.08 | 69.90 |
| 200 | ZS/ZF | 5.11 | 26.38 | 19.94 | 36.11 |
| 300 | ZS/ZF | 6.80 | 27.33 | 22.88 | 33.43 |
| 400 | ZS/ZF | 12.28 | 29.42 | 26.02 | 53.98 |
| 100 | ND-ZS/ZF | 3.62 | 0.01 | 3.33 | 27.12 |
| 200 | ND-ZS/ZF | 1.44 | 0.22 | 1.00 | 8.76 |
| 300 | ND-ZS/ZF | 2.02 | 0.68 | 1.66 | 7.10 |
| 400 | ND-ZS/ZF | 1.61 | 1.46 | 1.16 | 5.86 |

**References**

[1] N. Marzari, A. Ferretti, C. Wolverton, *Nat. Mater.* **2021**, 20, 736.

[2] G. Kresse, J. Furthmüller, *Comput. Mater. Sci.* **1996**, 6, 15.

[3] F. Turkakar, G, *J. Heat Transf.-Trans. ASME*. **2021**,143, 122901.

[4] H.A. Hasan, H. Togun, A.M. Abed, N. A. Qasem, H. Mohammed, A. Abderrahmane, K. Guedri, E.M. Tag-ElDin, *Symmetry-Basel.* **2023**, 15, 640.

[5] S. Z. Li, M. Xu, K. Chen, Q. Wu, Y. Li, C. Xie, Y. Li, Q. Xu, J. Huang, H. Xie, J. Colloid. *Interface. Sci.* **2024**,678, 1095.

[6] C. Fu, Y. P. Wang, C. Lu, S. Zhou, Q. He, Y. Hu, M.Y. Feng, Y.L. Wan, J.D. Lin, Y. F. Zhang, C. *Energy Storage Mater.* **2022**, 51, 588.

[7] P. Lin, J. Cong, J. Li, M. Zhang, P. Lai, J. Zeng, Y. Yang, J. Zhao, *Energy Storage Mater.* **2022**, 49, 172-180.

[8] T. T. Wei, Y. Ren, Z. Li, X. Zhang, D. Ji, L. Hu, *Chem. Eng. J.* 434, (**2022**) 134646.

[9] M. Q. Zhu, X. Li, C. W. Shi, C. Y. Cai, J. D. Zhang, *J Energy Storage.* **2024**, 101, 113686.

[10] W. J. Chen, Y. Tan, C. Y. Guo, X. Y. Zhang, X. He, W. Kuang, H. F. Weng, H. Du, D. Huang, Y.P. Huang, *J. Colloid. Interface. Sci.* **2024**,669, 104.
